# Novel Insights into the Bovine Polled Phenotype and Horn Ontogenesis in *Bovidae*

**DOI:** 10.1371/journal.pone.0063512

**Published:** 2013-05-22

**Authors:** Aurélie Allais-Bonnet, Cécile Grohs, Ivica Medugorac, Stefan Krebs, Anis Djari, Alexander Graf, Sébastien Fritz, Doris Seichter, Aurélia Baur, Ingolf Russ, Stéphan Bouet, Sophie Rothammer, Per Wahlberg, Diane Esquerré, Chris Hoze, Mekki Boussaha, Bernard Weiss, Dominique Thépot, Marie-Noëlle Fouilloux, Marie-Noëlle Rossignol, Este van Marle-Köster, Gunnfríður Elín Hreiðarsdóttir, Sarah Barbey, Dominique Dozias, Emilie Cobo, Patrick Reversé, Olivier Catros, Jean-Luc Marchand, Pascal Soulas, Pierre Roy, Brigitte Marquant-Leguienne, Daniel Le Bourhis, Laetitia Clément, Laura Salas-Cortes, Eric Venot, Maëlle Pannetier, Florence Phocas, Christophe Klopp, Dominique Rocha, Michel Fouchet, Laurent Journaux, Carine Bernard-Capel, Claire Ponsart, André Eggen, Helmut Blum, Yves Gallard, Didier Boichard, Eric Pailhoux, Aurélien Capitan

**Affiliations:** 1 Institut National de la Recherche Agronomique, UMR 1198 Biologie du Développement et Reproduction, Jouy-en-Josas, France; 2 Institut National de la Recherche Agronomique, UMR1313 Génétique Animale et Biologie Intégrative, Jouy-en-Josas, France; 3 Chair of Animal Genetics and Husbandry, Ludwig-Maximilians-University Munich, Munich, Germany; 4 Laboratory for Functional Genome Analysis, Gene Center, Ludwig-Maximilians-University Munich, Munich, Germany; 5 Institut National de la Recherche Agronomique, Plateforme bioinformatique Genotoul, UR875 Biométrie et Intelligence Artificielle, Castanet-Tolosan, France; 6 National Association of Livestock & Artificial Insemination Cooperatives, Paris, France; 7 Tierzuchtforschung e.V. München, Grub, Germany; 8 GeT-PlaGe, Genotoul, Castanet-Tolosan, France; 9 Institut National de la Recherche Agronomique, UMR444 Génétique Cellulaire, Castanet-Tolosan, France; 10 Institut de l’Elevage, Paris, France; 11 Genetic Analysis Laboratory for Animal Species, Jouy-en-Josas, France; 12 Department of Animal & Wildlife Sciences, University of Pretoria, Pretoria, South Africa; 13 Farmers Association of Iceland, Reykjavik, Iceland; 14 Institut National de la Recherche Agronomique, UE0326 Domaine expérimental du Pin-au-Haras, Exmes, France; 15 Gènes Diffusion, Douai, France,; 16 Evolution, Rennes, France; 17 UCATRC, Lempdes, France; 18 UALC, Naves, France; The University of Tennessee Health Science Center, United States of America

## Abstract

Despite massive research efforts, the molecular etiology of bovine polledness and the developmental pathways involved in horn ontogenesis are still poorly understood. In a recent article, we provided evidence for the existence of at least two different alleles at the *Polled* locus and identified candidate mutations for each of them. None of these mutations was located in known coding or regulatory regions, thus adding to the complexity of understanding the molecular basis of polledness. We confirm previous results here and exhaustively identify the causative mutation for the Celtic allele (P_C_) and four candidate mutations for the Friesian allele (P_F_). We describe a previously unreported eyelash-and-eyelid phenotype associated with regular polledness, and present unique histological and gene expression data on bovine horn bud differentiation in fetuses affected by three different horn defect syndromes, as well as in wild-type controls. We propose the ectopic expression of a lincRNA in P_C_/p horn buds as a probable cause of horn bud agenesis. In addition, we provide evidence for an involvement of *OLIG2*, *FOXL2* and *RXFP2* in horn bud differentiation, and draw a first link between bovine, ovine and caprine *Polled* loci. Our results represent a first and important step in understanding the genetic pathways and key process involved in horn bud differentiation in *Bovidae*.

## Introduction

What is more natural for cattle than to have horns? Horns, made of a pneumatized bony core fused with the skull frontal bone and covered by a continually growing keratin sheath, are a distinctive feature of the *Bovidae* family. However, these appendages have become undesirable in the modern cattle industry. Human and animal safety, economic losses due to horn injuries [1], [2] and the use of headlock feeding barriers are all reasons that led to the widespread practice of cattle dehorning during the last century. Because of animal welfare concerns [3], there has been a growing emphasis in recent years on breeding genetically hornless (i.e., polled) cattle to provide a non-invasive and long-term solution to this problem. Documented throughout history since ancient Egypt [4], [5], this autosomal dominant phenotype [6]–[10] was the first bovine locus studied after the rediscovery of Mendel’s Laws of Heredity, and has been the subject of massive research efforts in the last 20 years. While the *Polled* mutation has been easily mapped on bovine chromosome 1 (BTA01) in more than ten breeds [11]–[17], fine characterization of this locus has proved more difficult than expected and, to date, neither the causal mutation(s) nor the molecular etiology of this phenotype have been definitively identified. Among the major issues encountered in this process are: (i) the lack of appropriate (i.e., horned) model species, which make it impossible to identify functional candidate genes from previous studies; (ii) the absence of candidate polymorphisms in the coding sequences of any of the positional candidate genes [18]–[20]; and (iii) the absence of differential expression of the same genes between horn buds from polled and horned newborn calves [21].

After Identity-by-descent (IBD) mapping of the *Polled* locus based on Illumina Bovine50SNP beadchip genotyping data [22], we used targeted capture sequencing, to identify candidate causative mutations without relying on any *a priori* assumptions of gene function. This initial study provided evidence for the existence of at least two different alleles at the *Polled* locus (one “Celtic”, P_C_, and one “Friesian”, P_F_) and identified one and five candidate mutations, respectively, for each of them [19]. Intriguingly, none of these mutations was located in known coding or regulatory regions, thus adding to the complexity of understanding the molecular basis of polledness.

In parallel we gained further insights into the gene pathways involved in horn ontogenesis by identifying mutations/genes associated with novel horn development defects. The Polled and Multisystemic Syndrome (PMS) and the Type 2 Scurs Syndrome (T2SS) were described, and their causative mutations highlighted the critical role played by *ZEB2* and *TWIST1* genes, two master regulators of epithelial-to-mesenchymal transition (EMT), in bovine horn bud differentiation [20], [23]. Nevertheless, we were not able to find a direct link between these two genes and any of the genes located in caprine (*FOXL2*, *PISRT1* and *PFOXic*) [24], [25], ovine (*RXFP2*) [26] or bovine (*IL10RB*, *IFNAR2*, *OLIG1* and *OLIG2*) [19], [22] *Polled* regions.

The purpose of this study was: (i) to confirm the results obtained by targeted resequencing [19] using exhaustive approaches; and (ii) to compare different horn defects to get better insights into the molecular mechanisms involved in polledness and horn ontogenesis.

## Results and Discussion

### A Loss of Genetic Diversity in Western European Cattle Breeds Prevents Reliable IBD Mapping of the *Polled* Locus

**Figure 1:**
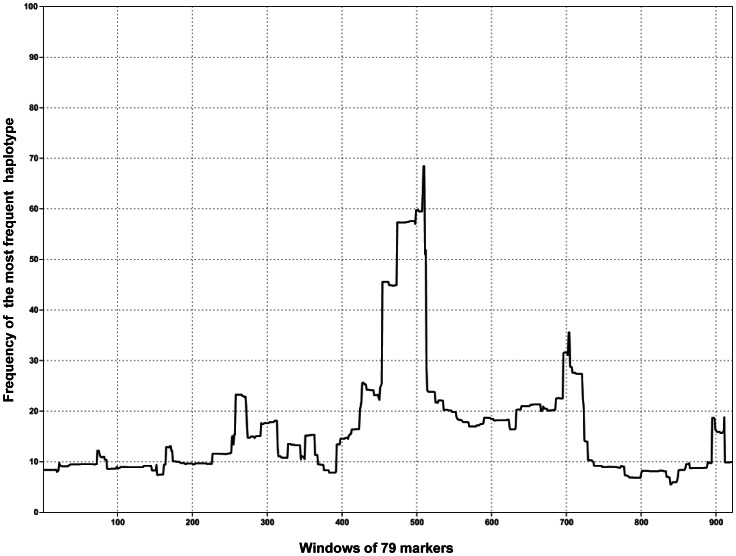
Frequency of the most frequent haplotypes across 17 Western European breeds for sliding windows of 79 markers at the beginning of BTA01. Only windows located in the first three megabases of BTA01, according to the UMD3.1 bovine genome assembly, are shown. Window 509, for which the maximal frequency is found (68.48%), corresponds to the segment located between positions 1,693,164 and 2,018,403.

In an independent attempt to fine map the *Polled* locus, we genotyped a panel of 51 animals (15 homozygous polled and nine paternal halfsib designs with a heterozygous founder) from nine different breeds, using the Illumina bovine 777K SNP beadchip. After phasing, we identified a large segment of 79 markers located at the beginning of BTA01 that was shared by all polled animals (ranging from position 1,693,164 to 2,018,403, according to the UMD3.1 bovine genome assembly [27]). To test the significance of this result, we then calculated the frequency of the same haplotype in 17 Western European horned breeds, based on analogous genotyping data from 4,843 animals. Surprisingly, the frequency of the “polled” haplotype was extremely high in all horned breeds (ranging from 44.3% in Rouge-des-Prés, to 88.5% in Salers), thus confirming the assumptions of Seichter et al. [22]. To measure the extent of the loss of genetic diversity in this region, we calculated haplotype frequencies within each breed for sliding windows of 79 markers along the first three megabases of BTA01. For each haplotype of each window, we then calculated an across-breed normalized frequency by adding the frequencies in the different horned breeds and dividing the sum by 17 (i.e., the total number of breeds). Finally, for each window, we used the normalized frequency of the most frequent haplotype as a proxy for evaluating genetic diversity in this region. Whereas the mean frequency of the most frequent haplotype for each window was only 16.35+/−11.38%, the “polled haplotype” showed the maximal normalized frequency among all the windows studied - and as a consequence, the least genetic diversity - with 68.48% (i.e., larger than the mean plus four standard deviations) (Fig. 1). These results support the existence of a selective sweep that occurred in Western European breeds long before the occurrence of the *Polled* mutation. By introducing a bias in IBD mapping of the *Polled* locus, such a selective sweep may have had serious consequences on previous results [19], and may have led to the sequence capture of a wrong interval.

### Accurate Mapping of the *Polled* Locus in Holstein and Charolais, Based on Observed Recombination Events

**Figure 2:**
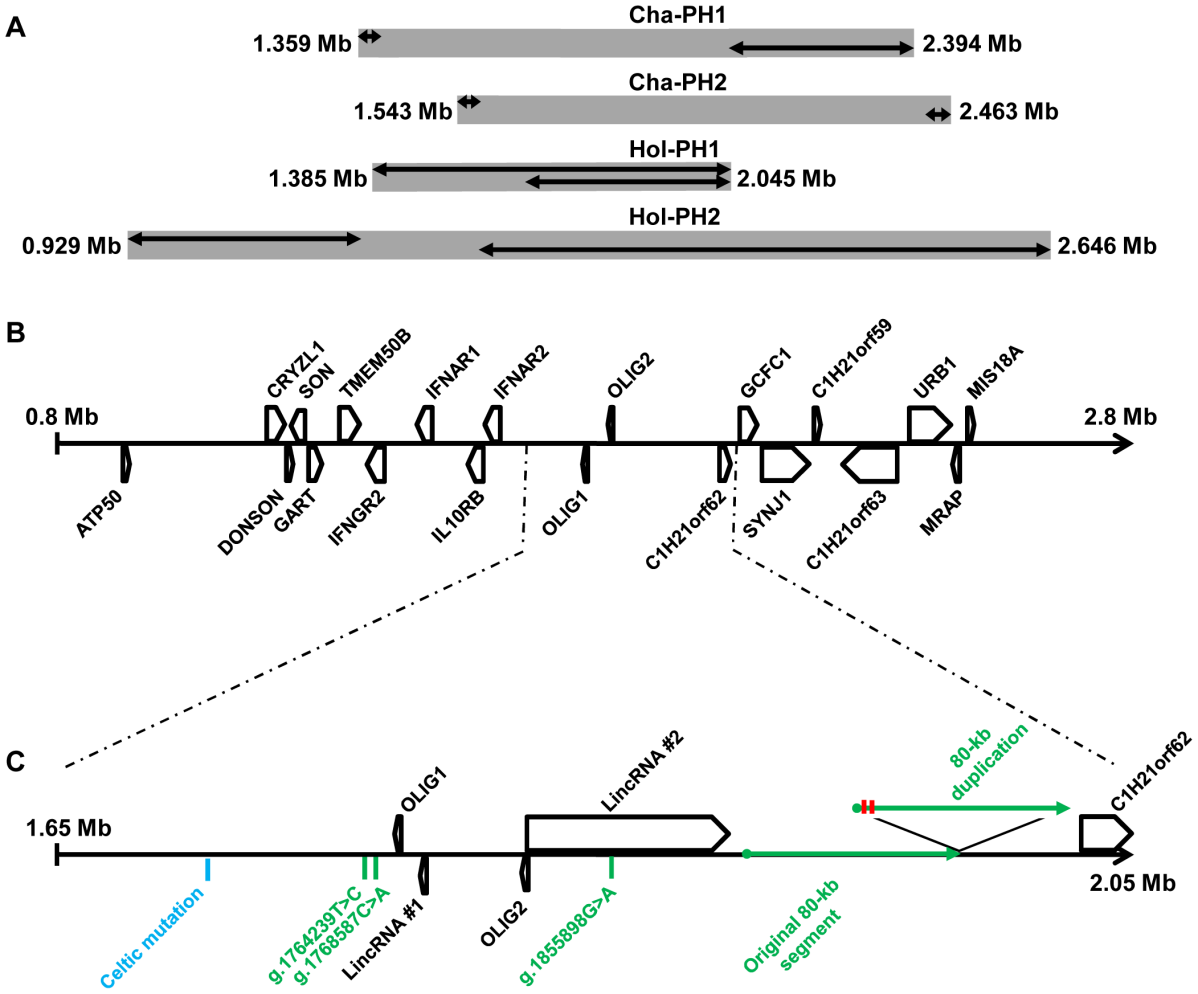
Accurate mapping of the *Polled* locus and identification of candidate causative mutations. (A) Localization on BTA01 of the intervals (gray boxes) containing the *Polled* mutations associated with different haplotypes based on Illumina BovineSNP50 beadchip genotyping data. Upper and lower double arrows indicate the region in which the most informative recombination occurred at the left and right border of the interval, respectively. (B) Gene content of the *Polled* intervals. (C) Localization of the candidate mutations for the Celtic (blue) and Friesian (green) *Polled* alleles, and details of the coding and non-coding genes in their close vicinity. LincRNA: Long intergenic non-coding RNA. LincRNA#1 and 2 correspond, respectively, to EST sequences n° AW356369, AW357421, BF654718, BM105296, BM254775, BM254845 and BC122836, DT831326, DT837875, DY200702, DY169884, EH130782, EH138227, EV606908, EV693397 in Genbank. Positions on BTA01 are based on the UMD3.1 bovine genome assembly. Polymorphism g.1855898G>A is located within an intron of LincRNA#2. Red bars indicate the two sequence variations between the original and the duplicated 80-kb segments.

To avoid any bias in mapping the *Polled* locus, we decided to investigate the French genomic selection dataset for recombination events. This dataset comprised haplotyping data from 3,349 Charolais and 76,851 Holstein animals (out of which 535 and 1,493, respectively, were born to at least one polled parent) that were genotyped with the Illumina BovineSNP50 beadchip. Analyzing BTA01 haplotypes from five Charolais and eight Holstein polled founders, we identified four different large haplotypes (two in each breed) that were never found among 189 and 1,233 horned founders, respectively. These consisted of 160, 191, 59 and 410 consecutive marker blocks (7.5, 9.8, 2.5 and 24.5 MB) encompassing previously reported locations of the *Polled* locus (not shown) [16]. Studying the descendants of these polled founders, we observed a total of 26, 28, 23 and 35 recombination events for Charolais polled haplotypes one (Cha-PH1) and two (Cha-PH2), and Holstein polled haplotypes one (Hol-PH1) and two (Hol-PH2), respectively. After obtaining phenotypic information for most of the carriers of recombining haplotypes, we were able to accurately map the *Polled* mutations associated with each of these haplotypes (Fig. 2A).

### Whole Genome Sequencing Data Confirm Allelic Heterogeneity of the Polled Phenotype

To exhaustively identify candidate causative mutations, we then sequenced the complete genome of two animals that were homozygous for the most frequent polled haplotype in their respective breeds (i.e., Cha-PH1 and Hol-PH1) using 100-bp paired-end reads. The average sequence depth of non-repetitive sequences within the corresponding polled intervals was 15.3x and 25.2x for Charolais and Holstein homozygous polled animals, respectively. From these data, we identified 1,610 and 1,225 putative DNA sequence variants in each polled interval, based on a comparison with the UMD3.1 genome assembly (which was obtained by sequencing a horned Hereford cow [28]). Filtering for variants found among analogous sequencing data from 55 horned bulls (one Charolais, eight Montbéliarde, nine Normande and 37 Holstein), we reduced the number of candidate causative mutations to 12 for Cha-PH1 and 47 for Hol-PH1 (Table S1). No variants were shared between both lists, thus confirming allelic heterogeneity of polled phenotypes associated with Cha-PH1 and Hol-PH1, which retrospectively corresponded to the previously reported Celtic and Friesian alleles [19].

### Different Genotyping Strategies Reduce the Number of Candidate Mutations for the Friesian and Celtic Allele to Four and One Polymorphisms, Respectively, Located in Non-Coding Regions

To further eliminate candidate mutations for the Friesian allele and refine the localization of the recombination points, we genotyped eight of the 47 candidate polymorphisms in animals carrying recombinant Hol-PH1 haplotypes, using a dichotomous approach. By doing this, we reduced the list of candidate mutations for this allele to three SNP (g.1764239T>C, g.1768587C>A, g.1855898G>A) and a large duplication of 80,128 bp (g.1909352_1989480dup), showing two sequence variations compared to the original segment, corresponding to g.1909354T>A and g.1909390_1909391del. Two of these polymorphisms (g.1768587C>A and the 80-kb duplication) belong to the five remaining candidates for the Friesian allele reported by our German group [19], whereas g.1764239T>C and g.1855898G>A had previously not been detected because of insufficient coverage. Since five small gaps in the UMD3.1 assembly and three regions with null coverage were observed in WGS data from the P_F_/P_F_ animal within the Friesian interval (between positions 1684055 and 2028953), these regions were subsequently sequenced in the same animal and three horned controls, using Sanger's method (Document S1, not shown). No novel candidate polymorphism was found, thus confirming the exhaustiveness of candidate polymorphisms reported for the Friesian allele.

To identify the causative mutation for the Celtic allele, we genotyped the 12 candidate polymorphisms in 32 homozygous polled animals from seven breeds belonging to the related Nordic and British breed groups. Among them, only one polymorphism showed complete concordance with the known genotype at the Polled locus: a duplication of 212 bp replacing a segment of 10 bp (g.1706051_1706060 delins 1705834_1706045 dup, hereinafter referred to as “P_C_” or “the Celtic mutation”), which was also the only remaining candidate polymorphism in the precedent non-exhaustive design [19]. To ensure the completeness of our research, we then compared WGS data from our P_C_/P_C_ Charolais bull with targeted sequencing data from four P_C_/P_C_ bulls [19] to determine the smallest IBD fragment shared between these animals around the Celtic mutation, and verified that no gap in the genome assembly or region with null coverage was found in our WGS data within this 8.6-kb long region (between positions 1700239 and 1708819).

Interestingly, neither the Celtic mutation nor the four remaining candidates for the Friesian allele are located within known genes or expressed sequence tags (Figs. 2B, 2C).

### Genotyping of a Large Panel of Animals Shows a Perfect Association Between the Polled Phenotype and the Celtic or Friesian Candidate Mutations

As an additional control, we genotyped a large panel of 2,732 polled and horned animals from 58 different European, African and Asian bovine breeds, and found a perfect association between the Friesian haplotype (defined by the candidate mutations for this allele) and/or the Celtic mutation with the polled phenotype (Table S2). Interestingly, segregation of the complete Friesian haplotype (∼320 kb) in Holstein and in a reduced number of horned breeds that were introgressed with polled genetics during the last century points to a recent occurrence of the Friesian mutation. On the contrary, fixation of the Celtic mutation in ancient Nordic and British breeds and segregation of this allele in Icelandic cattle, which evolved independently from other Nordic breeds since the time of the Vikings, makes it possible to date back this mutation to at least one millenary. Because of historical relationships between the Vikings and the ancient Germans and between the ancient Germans and the Scythians, this mutation could be even older and explain the polledness of the cattle raised by these people [29], [30], which Herodotus, in the fifth century BC, mistakenly attributed to the extreme cold of the modern day Ukrainian steppes. While tens of thousands of mutations affecting horn development must have occurred in horned populations since that time (based on the 63 cases of spontaneous mutations that we identified in French bovine populations in the last ten years; Capitan et al., unpublished data), it is intriguing to note that only the Celtic and Friesian mutations have been transmitted through the generations until today. This suggests that, contrary to bovine T2SS, bovine PMS and caprine Polled Intersex Syndrome (PIS), both mutations only affect horn ontogenesis and no other developmental processes. This thus would suggest that the *Polled* locus may be a *Bovidae*-specific gene.

### Different Evidence Suggests That the *Polled* Mutations Do Not Affect the Expression of a Bovidae-Specific Gene

To ascertain that the Celtic and Friesian polled alleles only affect horn development, we surveyed more than 100 French breeders, agricultural engineers and agricultural technicians for putative observation of additional phenotypes associated with polledness. Surprisingly, multiple occurrences of two phenotypes were reported: defects of the genital tract (“preputial eversion”, “pending prepuce”, “abnormal preputial withdrawal”, etc.) and atypical eyelashes (“bushy eyelashes”, “double rows of eyelashes”, etc.). We therefore investigated these phenotypes in polled and horned animals to characterize them. Visual examination of reproductive tracts from 42 polled and 69 horned bulls during semen collection revealed that after erection, both P_C_/P_C_ Charolais bulls and most of the P_C_/p Charolais bulls (11/14) displayed passive preputial withdrawal following penis withdrawal (Fig. S1). At rest, the same animals did not show preputial eversion or other detectable external defects, but according to technicians, they displayed absence of prepuce tonicity, making it very difficult to introduce a catheter in their sheath to perform conventional sheath cleaning. In contrast, none of the other Celtic polled, Friesian polled or horned animals examined showed similar symptoms, indicating that this phenotype, which is consistent with the absence of the retractor muscle of the prepuce previously reported in P_C_/P_C_ Galloway and Aberdeen-Angus bulls [31], is not completely associated with the Celtic mutation. It is more likely specified by another locus in moderate linkage disequilibrium with the Celtic mutation or by other breed-specific loci interacting with the *Polled* loci.

**Figure 3:**
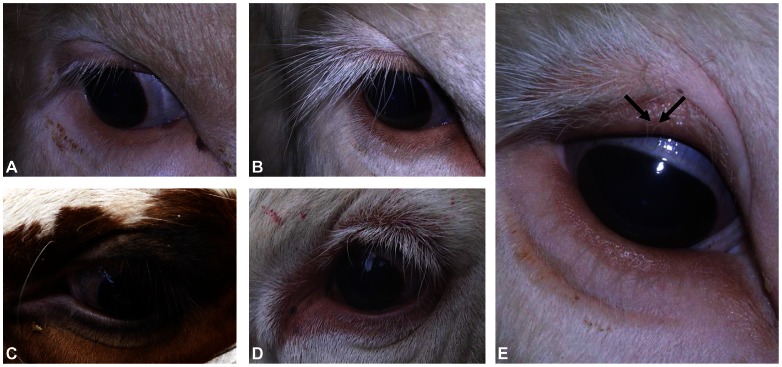
Details of the eyelash and eyelid phenotypes associated with polledness. Eyes of a wild-type bull (A), three P_C_/p Charolais bulls (B, D and E), and a P_F_/p Holstein cow (C). Note the typical eyelid hypertrichosis and accessory rows of lashes in polled animals, with additional hair showing pronounced variability in size and orientation and darker coloration in pigmented animals (C). Arrows show additional hair growing on the inner part of the eyelid (i.e., distichiasis).

On the contrary, visual examination of 166 horned and 78 polled animals confirmed a perfect association between polledness (whether of Celtic or Friesian origin) and an atypical eyelash-and-eyelid phenotype, thus revealing that the *Polled* locus does not only affect horn ontogenesis. In most cases, this phenotype was characterized by accessory rows of eyelashes and eyelid hypertrichosis, with additional hair showing unequal length, irregular orientation and darker coloration in pigmented animals (Figs. 3A–3C). In rare cases (less than 10%), the abnormal phenotype was milder and either restricted to additional rows of eyelashes of normal length (Fig. 3D) or to a single row of eyelashes with irregular hair length (not shown). No notable difference was found between Celtic or Friesian polled animals, indicating that both mutations have exactly the same phenotypic consequences. Finally, closer examination of one P_C_/P_C_ Charolais bull with a particularly pronounced phenotype showed additional hair growing on the inner part of the eyelid, i.e., distichiasis (Fig. 3E).

To our knowledge, only two genes have been associated with comparable eyelash phenotypes until now and, more precisely, with dominant distichiasis in humans: *FOXC2* [32], [33] and *TWIST2* [34]. While neither of them is located on chromosome one in bovines, it is striking to note that these genes are paralog to *FOXL2* and *TWIST1*, respectively, which are associated with goat PIS [24] and bovine T2SS [23]. Moreover, they both encode transcription factors that are able to promote EMT [35]–[37] like *TWIST1* and *ZEB2*, which is associated with a third horn defect syndrome in bovines: PMS [20]. Finally, complete deficiency of *TWIST2* in humans and mice causes bitemporal skin lesions (characterized by hypoplastic dermis and the absence of subcutaneous fat and epidermal appendages) [38], unraveling an evolutionary conserved cryptic expression of this gene in mammals at the exact anatomical location of horns in *Bovidae* and constituting an additional argument in favor of a possible role of *TWIST2* in horn ontogenesis.

Taken together, these observations finally suggest that the polled alleles do not rely on a previously unknown *Bovidae*-specific gene that would only be expressed in developing horn. They more likely affect the expression of genes involved in the differentiation of other skin appendages in mammals, i.e. eyelash and eyelid hair follicles that have acquired a role in horn ontogenesis during *Bovidae* evolution. The excellent conservation of the wild-type allele of the Celtic mutation between *Bovidae* and non-horned ruminants (Document S2) and the existence of 30 highly conserved regions between eutherian mammals within the Friesian 80-kb duplicated segment (between 5 and 790 bp, according to the Ensembl conservation track [39]), underlying the existence of an evolutionary conserved role of these non-coding regions prior to the appearance of horns in *Bovidae*, also support this hypothesis.

To further investigate the consequences of the Celtic mutation on horn bud differentiation, we then produced seven P_C_/p fetuses and seven wild-type controls (wt) at 90 dpc (days *post-coïtum*) before performing histological and gene expression analyses. Four 90-dpc fetuses affected by previously characterized horn defect syndromes (two PMS, heterozygous for a 3.7-Mb deletion encompassing *ZEB2,* and two T2SS, heterozygous for a frame shift mutation in the beginning of *TWIST1*) were also included in the study for the purpose of comparison (see below).

### Histological Analysis Reveals an Absence of Horn Bud Differentiation in P_C_/p 90-dpc Fetuses

**Figure 4:**
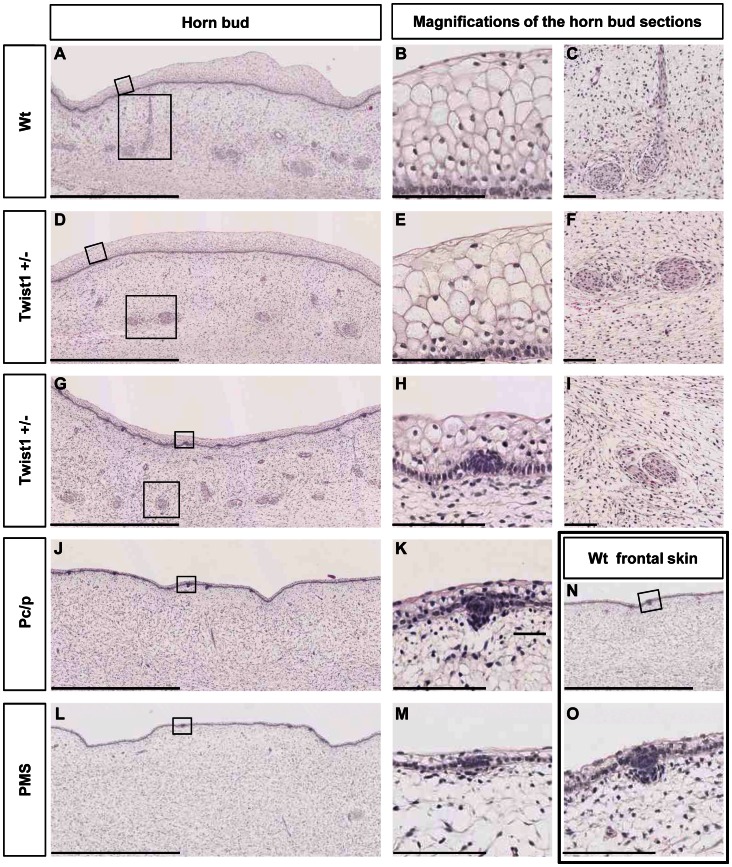
Histological analyses of horn buds and forehead skin from wild-type fetuses and fetuses affected by different horn-defect syndromes. (A), (D), (G), (J) and (L) Histological sections of horn buds of a wt, two TWIST1+/−, a P_C_/p and a PMS fetus, respectively. (B) and (C), (E) and (F), and (H) and (I) Magnifications (X10 and X3, respectively) of (A), (D) and (G). (K) and (M) Magnifications (X10) of (J) and (L). (N) Histological section of the forehead skin of a wt fetus. (O) Magnification (X10) of (N). Scale bars in (A), (D), (G), (J), (L) and (N) represent 1 mm, whereas scale bars in (B), (C), (E), (F), (H), (I), (K), (M) and (O) represent 100 µm.

As previously observed [20], histological sections of wt horn buds were characterized by three main features as compared with wt frontal skin: clusters of dermal cells displaying glandular/ductal differentiation, supernumerary layers of vacuolated keratinocytes and absence of hair follicle germs (Figs. 4A–4C, 4N and 4O). In *TWIST1*+/− fetuses, the anatomy of horn buds from one individual was essentially identical to that of wt horn buds (Figs. 4A–4F) and only differed from them by displaying flatter “keratin sheath primordium” (Figs. 4A, 4D), whereas horn buds from the other individual were characterized by an intermediate phenotype between wt horn buds and forehead skin (Figs. 4G–4I). On the contrary, horn buds from P_C_/p and PMS fetuses did not show anatomical differences with their respective forehead skin. However, while tissues from P_C_/p fetuses were apparently identical to wt forehead skin (Figs. 4J, 4K, 4N and 4O), tissues from PMS fetuses were characterized by reduced hair follicle germ sizes and a significantly thinner epidermis (Figs. 4L–4O and [20]).

In summary, at 90 dpc, horn buds from fetuses affected by the three genetic abnormalities studied showed defects consistent with symptoms observed in adulthood (i.e., abnormal horn in T2SS and absence of horn growth in polled and PMS animals). As previously reported for PMS [20], regular polledness and T2SS phenotypes are thus caused by impaired or abnormal differentiation of the horn bud during embryogenesis and not by a defective stimulation of this organ after birth. However, contrary to PMS, deleterious effects of the Celtic mutation and of *TWIST1* loss of heterozygosity seem to be mainly limited to the horn bud area since no particular feature was observed in the frontal skin.

### RT-qPCR Analysis Identifies the Overexpression of a LincRNA in P_C_/p Horn Buds as a Credible Cause of Horn Bud Agenesis

**Figure 5:**
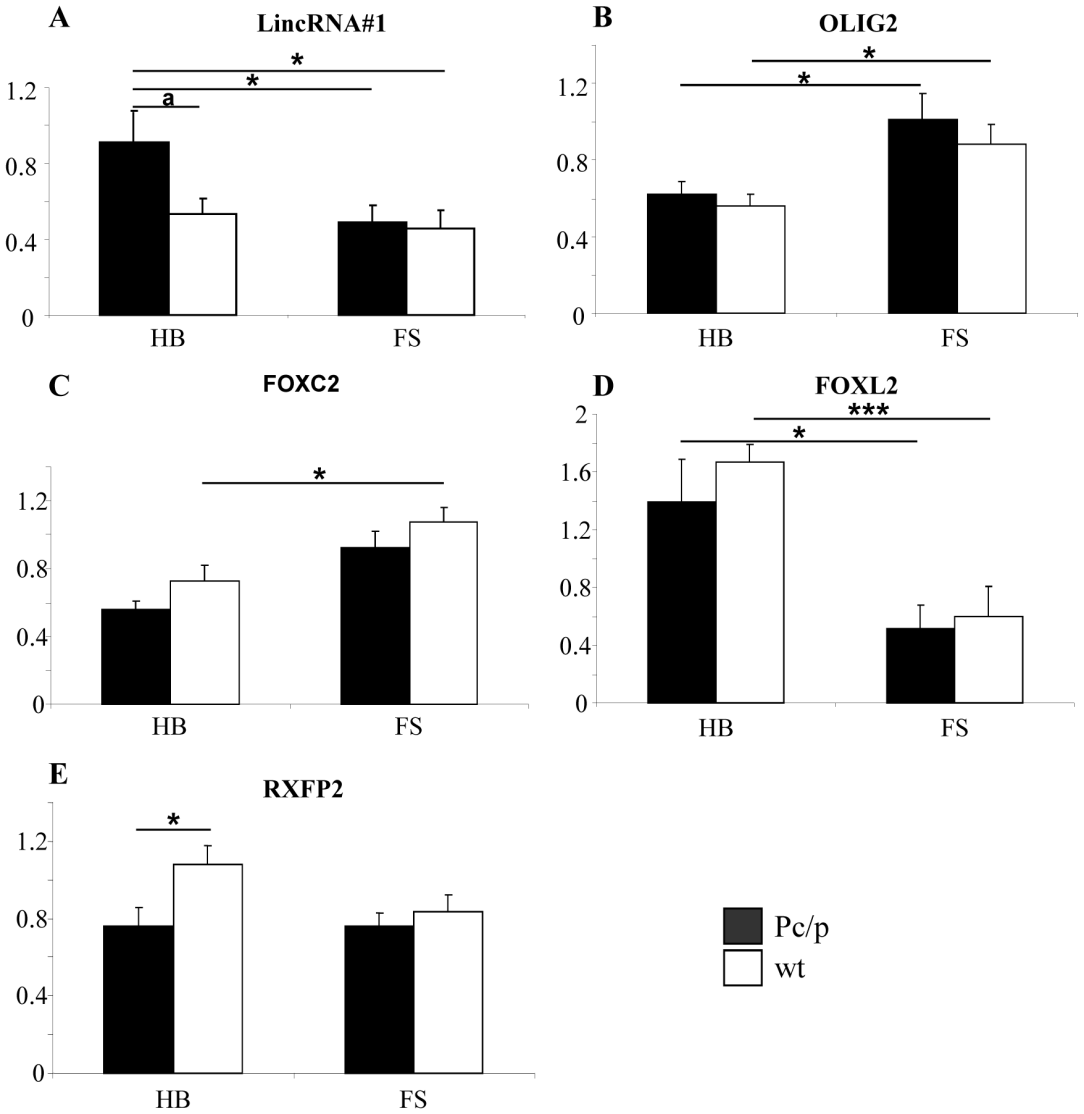
RT-qPCR gene expression analyses in tissues from wild-type and P_C_/p 90 dpc fetuses. HB: horn buds+frontal bone, FS: frontal skin+frontal bone. *p<0.05, **p<0.01, ***p<0.001, and a: p = 0.052 (Fischer’s test).

Using Reverse Transcription quantitative PCR (RT-qPCR), we then analyzed the expression of protein coding genes located 500 kb up and downstream the Celtic mutation (i.e., *GART*, *TMEM50*, *IFNGR2*, *IFNAR1*, *IL10RB*, *IFNAR2*, *OLIG1*, *OLIG2*, *C1H21orf62*, *GCFC1*) in horn buds and frontal skin from seven P_C_/p and seven wt fetuses. Among them, only *OLIG2* showed significant differences in expression levels between at least two conditions and, more precisely, between horn buds and frontal skin in both genotypes (Fig. 5). This result suggests that *OLIG2* plays a role in horn ontogenesis and indicates that the Celtic mutation has no effect on its transcription since expression profiles were similar in P_C_/p and wt fetuses.

In a second attempt, we analyzed the expression of two LincRNA located between the Celtic mutation and the Friesian 80-kb duplication (see gene location in Fig. 2), and observed a tissue-specific overexpression of LincRNA#1 in P_C_/p horn buds with significant differences (p<0.05) vs. P_C_/p or wt frontal skin, and suggestive differences (p = 0.052) vs. wt horn buds. Since we had already experienced difficulties in observing significant differences in LincRNA expression in the heterozygous state [24] and since two independent RT-qPCR gave exactly the same expression profiles (with p = 0.100 and 0.078 between P_C_/p and wt horn buds; not shown), we believe that the latter difference in LincRNA#1 expression between P_C_/p and wt horn buds is not an artifact.

To the best of our knowledge, the regular function of this LincRNA, which displays some highly conserved exons among eutherian mammals (according to the Ensembl conservation track [39]) is still unknown. However, based on the absence of similar expression profiles among the genes studied and based on its location, it can be hypothesized that LincRNA#1 does not regulate the expression of neighboring genes, contrary to most of the LincRNA.

Thus, in the light of previous observations (suggesting that the *Polled* mutations affect regions regulating the expression of genes conserved with non-horned species and involved in developmental processes other than horn ontogenesis), tissue-specific overexpression of LincRNA#1 in P_C_/p horn buds is the most credible cause of horn bud agenesis at this time. Similar investigations in P_C_/P_C_ and P_F_/P_F_ fetuses, and identification and characterization of the targets of this LincRNA are now required to definitely confirm this hypothesis.

### EMT No Longer Plays a Role in Horn Ontogenesis at 90 dpc

Meanwhile, we investigated the role of other functional candidate genes in the same dataset to obtain better insights into the mechanism involved in normal and pathological horn bud differentiation. These comprised *FOXL2*, *RXFP2*, *TWIST1* and *ZEB2* (gene mapping to loci involved in horn defect syndromes in *Bovidae)*, *TWIST2* and *FOXC2* (involved in distichiasis in model species), and *E-Cadherin*, *N-Cadherin*, *Occludin* and *Vimentin* (four markers of EMT).

With the exception of *FOXC2* (Fig. 5), none of the master regulators of EMT (i.e., *TWIST1*, *TWIST2* and *ZEB2*) and none of the markers of this process showed differential expression in the tissues studied at this stage (not shown). From our point of view, these results do not rule out a possible role of EMT in horn ontogenesis *per se,* but focus instead on an earlier involvement of this process in the differentiation of horn bud precursor cells, as suggested by the key role played by *TWIST1* and *ZEB2* (whose loss of heterozygosity is associated with T2SS and PMS horn development defects) in the differentiation of several types of precursor cells from the cranial neural crest [40]–[44]. In addition to *FOXC2,* two other genes showed differential expression between at least two tissues: *RXFP2* and *FOXL2* (Fig. 5). To confirm these results and investigate the expression of differentially expressed genes in the context of other pathological horn bud differentiation, we subsequently performed the same analysis on tissues from two *TWIST1*+/− and two PMS fetuses, as well as ten of the 14 fetuses previously studied (5 P_C_/p and 5 wt).

**Figure 6:**
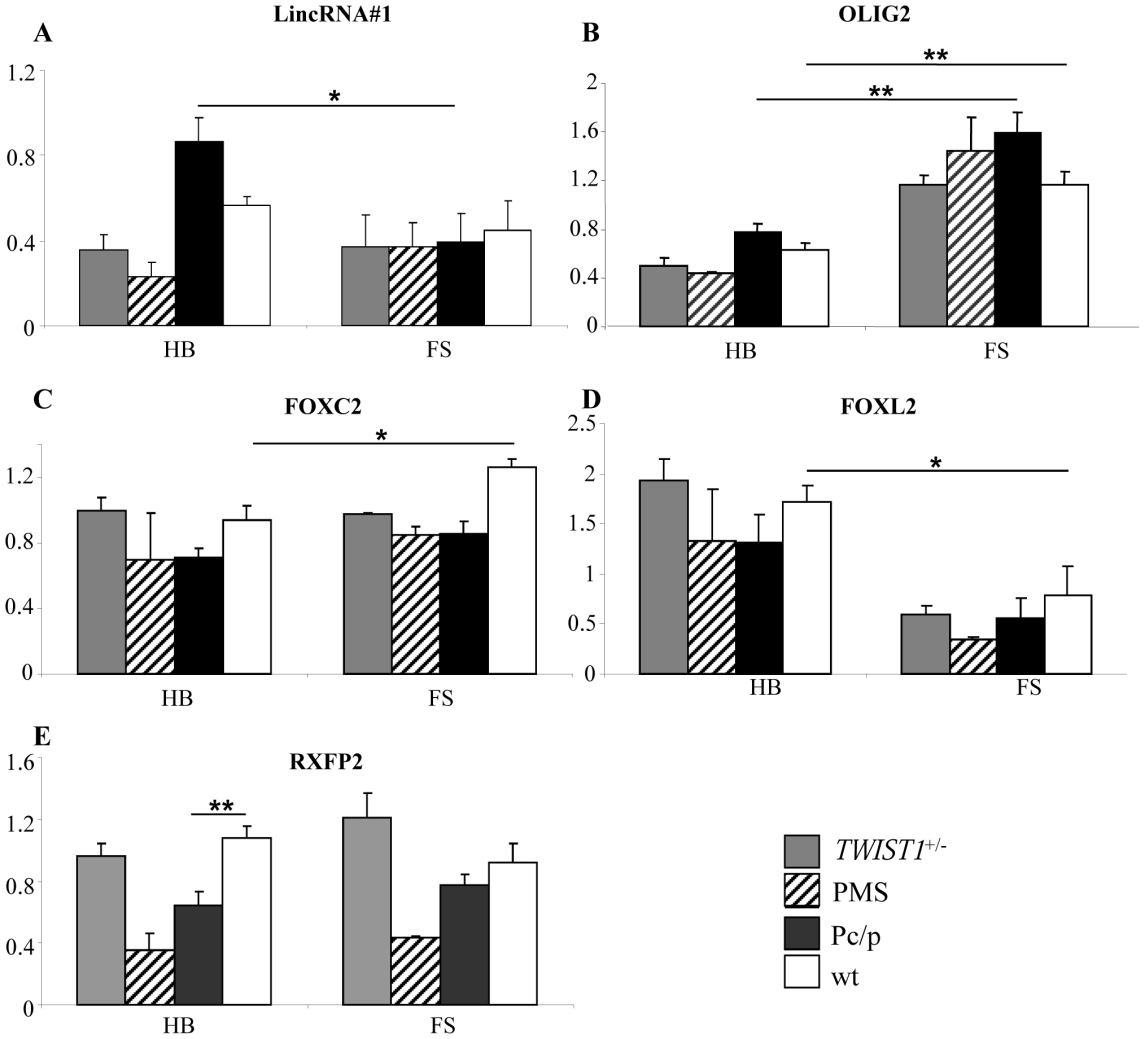
RT-qPCR gene expression analyses in tissues from wild-type, P_C_/p, *TWIST1*+/− and PMS 90 dpc fetuses. HB: horn buds+frontal bone, FS: frontal skin+frontal bone. *p<0.05 and **p<0.01 (Fischer’s test). P-values were not calculated for expression levels in tissues from TWIST1+/− and PMS fetuses due to the small number of individuals investigated.

By doing this, we confirmed a significant overexpression of *FOXC2* in wt frontal skin with significant differences vs. wt horn buds, and suggestive differences vs. tissues of different genotypes (of note, statistics were not calculated for *TWIST1*+/− and PMS fetuses due to the small number of individuals). Present knowledge did not enable us to find credible biological explanations for this result, contrary to those of LincRNA#1, *OLIG2*, *TWIST2* and *RXFP2* gene expression analysis (Fig. 6).

### LincRNA#1 is not Overexpressed in Defective Horn Buds other than P_C_/p Horn Buds

Interestingly, RT-qPCR analysis suggested an absence of LincRNA#1 overexpression in tissues other than P_C_/p horn buds. The fact that this feature is specific to P_C_/p horn buds and is not a generic marker of abnormal horn bud differentiation gives further weight to the hypothesis that ectopic expression of this LincRNA would repress genes involved in horn bud differentiation and be the cause of regular polledness.

### *FOXL2* and *OLIG2* are Differentially Expressed between Frontal Skin and Horn Buds

Analyzing *FOXL2* and *OLIG2* expression, we confirmed previous results and observed suggestive differences in the expression of these genes between horn buds and frontal skin, regardless of the genotype studied. Both showed antagonistic expression profiles with an overexpression of *FOXL2* and an underexpression of *OLIG2* in horn buds versus frontal skin. These results support a role of both genes in horn ontogenesis and indicate that defective horn bud differentiation does not prevent expression of some gene pathways involved in horn ontogenesis. As a consequence, they indicate that while histological analysis did not show visible differences between horn buds and frontal skin from P_C_/p or from PMS fetuses, the non-differentiated horn buds are not similar to the regular frontal skin at the molecular level. These results also provide novel insights into the molecular mechanisms involved in the polled phenotype associated with goat PIS. Indeed, in previous experiments, we observed a significant overexpression of *FOXL2* in horn buds from PIS −/− vs. 70 dpc control fetuses [24] but, until now, we believed that horn bud agenesis was caused by the ectopic expression of this transcription factor. Taken together, observations made in goat and bovine suggest that *FOXL2* is involved in the negative regulation of horn bud differentiation (perhaps to control the diameter of the horn bud or to prevent horn growth before birth). Finally, while no difference in *OLIG2* expression was found between P_C_/p and wt horn buds, the probable involvement of *OLIG2* in horn bud differentiation combined with its genomic location provides a strong incentive for performing post-transcriptional analysis to investigate possible modifications of the regulation of this gene associated with the *Polled* mutations.

### Horn Bud Agenesis in P_C_/p and PMS Fetuses is Associated with Reduced Expression of *RXFP2*

RT-qPCR analysis confirmed the significant reduction of *RXFP2* expression previously observed in P_C_/p vs. wt horn buds and showed a pronounced reduction of RXFP2 expression in both PMS horn buds and frontal skin, compared to other tissues. The association of *RXFP2* underexpression with horn bud agenesis suggests that this gene plays a key role in horn bud differentiation. A reduced expression of this gene, which is the only gene mapping to the sheep *Polled* locus [26], seems therefore to be the most probable cause of polledness in this species since complete lack of function of *RXFP2* would lead to cryptorchidia in homozygous rams (see below). The fact that *TWIST1*+/− horn buds did not show a pronounced difference in *RXFP2* expression is not surprising since *TWIST1*+/− fetuses displayed abnormal but differentiated horn buds. Underexpression of *RXFP2* in both horn buds and frontal skin from PMS fetuses also suggests that this gene is positively regulated by ZEB2 and that it plays a yet unknown role in skin differentiation since PMS fetuses display a slight delay in skin differentiation, characterized by reduced epithelium thickness and hair follicle primordium size.

Although *RXFP2* expression has been demonstrated in various human and mouse tissues, including skin, the precise roles of this receptor in many of these tissues are unknown. Indeed, following the report of abnormal differentiation of the gubernaculae (ligaments that control testicular descent) and consequent intra-abdominal cryptorchidism in mice deficient in RXFP2 or in its testes-secreted ligand, INSL3 [45]–[48], these genes have generally been studied in the context of the development of the reproductive tract of male eutherian mammals. Investigation in other contexts has just begun and new roles of INSL3-RXFP2 signaling have recently been reported in kidney differentiation [49] and bone metabolism [50]–[52].

A possible involvement of RXFP2 (also known as LGR8) in skin differentiation, in general, and in horn bud differentiation, in particular, is also supported by the function of its second type of ligands, the relaxins. Relaxins modulate connective tissue remodeling in a wide range of tissues, including derma, by altering matrix molecule expression [53]–[56]. They possess antifibrotic properties and are successfully used in the treatment of skin fibrosis in humans [57]–[61]. In addition, other receptors belonging to the LGR (Leucine-rich repeat-containing G-protein coupled receptors) family and their ligands are also involved in skin differentiation. For example, in humans, complete deficiency in R-spondin-1 and R-spondin-4, two ligands of LGR4, LGR5 and LGR6 [62]–[68], are responsible for palmoplantar hyperkeratosis and predisposition to squamous cell carcinoma of the skin associated with XX sex reversal [69], and for the absence or hypoplasia of nails [70], respectively. Moreover, complete deficiency in RXFP1 (also known as LGR7) or in Relaxin-1, one of its ligands, causes abnormal nipple development in mice [71]–[73]. Finally, the fact that RXFP2 and its close relative, RXFP1, which are conserved among vertebrates, have been involved in the development of novel organs in the evolution of mammals (gubernaculae and nipples, respectively) makes them good candidates to participate in horn ontogenesis. If confirmed, the role of RXFP2 in this process may explain sexual dimorphism between male and female horns in *Bovidae*, through the action of testes-secreted INSL3. The increase of INSL3 serum concentration during male puberty [74] may also be responsible for the frequent appearance of keratin outgrowth in P_C_/p and P_F_/p bulls from the age of 8 to 12 months.

### Conclusions

In conclusion, using exhaustive approaches, we confirmed the allelic heterogeneity of the bovine *Polled* locus and identified one and four candidate mutations for the Celtic and Friesian alleles, respectively. We presented unique histological and gene expression data on bovine horn bud differentiation in fetuses affected by three different horn defect syndromes and in wild-type controls. In addition we described a previously unreported eyelash-and-eyelid phenotype associated with regular polledness, and provided ample evidence suggesting that the Polled mutations do not affect a Bovidae-specific gene but more likely cause ectopic expression in horn buds of a LincRNA conserved among mammals. Finally, we added *OLIG2*, *FOXL2* and *RXFP2* to the list of genes involved in horn ontogenesis and, for the first time, we found a link between, bovine, ovine and caprine Polled loci. Further studies are required to confirm part of the results reported here but this study undoubtedly marks an important milestone in understanding the genetic pathways and key processes involved in horn bud differentiation.

## Materials and Methods

### Ethics Statement

Experiments reported in this work comply with the ethical guidelines of the French National Institute for Agricultural Research (INRA) and the Ludwig-Maximilians-University (LMU) of Munich. Ninety *dpc* fetuses were produced on an INRA experimental farm in Le-Pin-au-Haras and collected in the SOCOPA slaughterhouse in Gacé, both located in the Orne Department (France). The protocol was approved by the Division of Social Cohesion and Protection of Populations of the Orne Department (DDCSPP 61), and Aurélien Capitan is the recipient of an official authorization for animal experimentation from the DDCSPP 61. No ethical approval was necessary for the eyelash and prepuce phenotype examinations or for blood and sperm sampling since they were performed during routine husbandry procedures. Blood was collected by veterinarians or by agricultural technicians licensed by the French Departmental Breeding Establishments (Etablissements Départementaux de l’Elevage (EDE)) during routine blood sampling for paternity testing, annual prophylaxis, or genomic selection purpose. Sperm was obtained from semen straws collected by approved commercial artificial insemination stations as part of their regular semen collection process. All the samples and data analyzed in the present study were obtained with the permission of breeders, breeding organizations and research group providers.

### Animals, Phenotyping and Sampling

All animals studied in this project are presented in Table S3 according to their breed or species affiliations and their purpose in the experimental design. DNA was either generously provided by research partners or was extracted from blood using the Wizard® Genomic DNA Purification Kit (Promega, Charbonnières-les-Bains, France) or from fetal liver and sperm using a standard phenol-chloroform method.

To avoid any confusion with other loci, only polled animals with well-documented pedigrees (information on at least five generations) and with either one healthy homozygous polled relative or polled ancestors with the same guarantee were sampled. To avoid confusion between polled and scurs or scurs and horned phenotypes, only animals that displayed clean polled phenotypes (i.e., without any evidence of horn growth) or horns firmly attached to the skull at the age of nine months or more were sampled and referred to as “polled” and “horned”. In France most of the calves belonging to polled breeding schemes are routinely examined twice for this phenotype (between four and six months and between nine and 18 months [75]), thus making *a posteriori* collection of phenotypes for individuals bearing recombining haplotypes possible. Eyelids and eyelashes were visually examined during milking, feeding and other routine husbandry procedures during which animals are motionless. Retraction of the prepuce was examined after a minimum of three false matings for each bull during semen collection in French and German-approved commercial artificial insemination stations.

Most of the fetuses were obtained by inseminating wt Charolais and Holstein X Normande crossbred cull cows with the semen of a *TWIST1+/−* Charolais bull (producing two *TWIST1+/−* females, one wt female and two wt males), of a P_C_/P_C_ Charolais bull (four P_C_/p females, three P_C_/p males), and of one wt Charolais bull (one wt male). In addition, two wt and two PMS female fetuses had previously been produced using *in vitro* fertilization and embryo transfer [20], [76]. All the fetuses analyzed in this study had a minimum of 50% of Charolais genetic stock. Pregnant cows were slaughtered on day 90 (stunning and subsequent bleeding) and the dead fetuses were recovered from their genital tracts. The right horn bud+frontal bone and right forehead skin+frontal bone were collected from each fetus for expression studies, whereas the left horn bud+frontal bone and left forehead skin+frontal bone were collected for histological analyses. Fetuses produced with the semen of heterozygous or mosaic bulls were genotyped as previously described [20], [23] to confirm their genotype.

### IBD Mapping of the *Polled* Locus

Fifty-one animals were genotyped with the Illumina bovine 777K SNP beadchip for this project and 4,843 within the framework of other projects led by the INRA GABI unit (minimum call rate per sample: 0.95). Markers with call rates lower than 0.90, deviating from the Hardy-Weinberg equilibrium within breeds, or which were monomorphic over the whole dataset were discarded. Quality control also included parentage checking in the small paternal half-sib design and elimination of remaining Mendelian inconsistencies. A total of 1,046 markers located within the first three megabases of BTA01 were included in the study. Haplotype phasing was then performed within each breed using BEAGLE 3.3.2 software [77]–[78] and setting the scale and shift parameters to 2 and 0.1, respectively. Finally, haplotypes were analyzed as described in the Results/Discussion section.

### Accurate Mapping of the *Polled* Locus using Illumina BovineSNP50 Beadchip Haplotyping Data from the French Genomic Selection Database

Illumina BovineSNP50 beadchip haplotyping data were extracted from the French database for Holstein and Charolais genomic selection. Briefly, the French genomic selection procedure [79] includes: (i) elimination of markers with minor allele frequency lower than 3% in each breed, deviating from the Hardy-Weinberg equilibrium or without a known position on the UMD3.1 genome assembly; (ii) parentage checking and elimination of remaining Mendelian inconsistencies; and (iii) phasing and imputation of some missing genotypes with DualPhase, part of the PhaseBook software [80]. All together, BTA01 haplotypes were composed of 2,466 and 2,863 markers along this chromosome in Charolais and Holstein, respectively. Because sires and maternal grand-sires of all animals are genotyped, these phases are highly reliable. As an additional security, only animals belonging to half-sib families with a minimum of ten progeny per sire were kept (i.e., 3,349 Charolais and 76,851 Holstein). Finally, only recombining haplotypes with a minimum of two carriers with accurate and concordant phenotypes were considered for the mapping of the *Polled* locus.

### Whole Genome Sequencing, Read Mapping and Variant Calling

Paired-end libraries with a 250-bp insert size were generated for one Holstein cow that was homozygous for Ho-PH1 and one Charolais bull that was homozygous for Ch-PH1 using the Illumina TruSeq DNA Sample Prep Kit. The two libraries were quantified using the QPCRLibrary Quantification Kit (Agilent), controlled on a High Sensitivity DNA Chip (Agilent) and sequenced on two HiSeq 2000 lanes (Illumina), each with Illumina TruSeq V3 Kit (200 cycles). The 100-bp reads were mapped on the UMD3.1 bovine sequence assembly using the BWA tool [81]. Only reads with a unique mapping and a minimal quality of 30 were kept. PCR duplicates were filtered and a pileup of the mapped reads was created for each animal using SAMtools [82]. SNP and small indels (up to 10 bp) were detected with the Genome Analysis Tool Kit 1.1–3 (GATK) [83], whereas the discovery of larger indels was achieved with Pindel [84]. Finally, detection of Copy Number Variation was performed according to Medugorac et al. [19]. Polymorphisms identified in each animal were then independently filtered for polymorphisms found in whole genome sequencing data from 55 horned bulls (one Charolais, eight Montbéliarde, nine Normande and 37 Holstein), and processed with the same tools within the framework of other projects led by the INRA GABI unit. The average sequence depth of non-repetitive sequences within the polled intervals ranged from 5.9x to 39.8x for these horned animals. Genome assembly gaps and regions with null coverage mapping to the restrained *Polled* intervals were PCR amplified and sequenced using the Sanger method, as described in the following section.

### Genotyping of the Candidate Causative Mutations

To refine the localization of the recombination point in recombining Friesian haplotypes, eight polymorphisms among the candidates were genotyped based on a dichotomous approach. The seven SNP (g.1654405G>A, g.1655463C>T, g.1684055G>C, g.1764239T>C, g.1768587C>A, g.1855898G>A and g.2028953C>G) were genotyped by PCR-Sequencing. PCR primers were designed from the UMD3.1 bovine genome assembly with Primer3 software [85]. The list of all the primers used in this study is available in Table S4. PCR reactions were performed using Go-Taq Flexi DNA Polymerase (Promega), according to the manufacturer’s instructions, on a Mastercycler pro thermocycler (Eppendorf). The resulting amplicons were purified and bidirectionally sequenced by Eurofins MWG (Germany) using conventional Sanger sequencing. Polymorphisms were detected with NovoSNP software [86]. The 80-kb duplication was genotyped by PCR-Electrophoresis. A 297-bp PCR product encompassing the junction between the two 80-kb segments and a 97-bp control region were amplified under the same conditions and size, separated by 2% agarose gel electrophoresis. This diagnostic test does not make it possible to distinguish the homozygous from the heterozygous carriers of the 80-kb duplication. However, this was not a problem since only heterozygous carriers of recombining haplotypes were analyzed at this step.

To identify the causative mutation for the Celtic allele, the 11 candidate SNP were genotyped by PCR-sequencing, whereas g.1706051_1706060 delins 1705834_1706045 dup polymorphism was genotyped by PCR-Electrophoresis, as previously described. For the latter diagnostic test, the wt and mutated alleles correspond to 379-bp and 581-bp fragments, respectively.

Finally, two different strategies were implemented to genotype the large panel of 2,732 animals for the Celtic mutation, the Friesian 80-kb duplication and the three other candidate mutations for the Friesian allele (SNP g.1764239T>C, g.1768587C>A and g.1855898G>A). A total of 631 animals were genotyped in France for these five mutations, as mentioned above. In the rare cases where animals were homozygous for the three candidate SNP alleles associated with the Friesian allele, another SNP (g.2028953C>G) situated on the opposite side of the 80-kb deletion and showing very strong linkage disequilibrium with the Friesian allele was genotyped to distinguish the homozygous from the heterozygous carriers of the 80-kb duplication animals. The other 2,101 animals were genotyped for the Celtic mutation, the Friesian 80-kb duplication, SNP g.1768587C>A, and two other polymorphisms (g.1654405G>A and g.1655463C>T) showing strong linkage disequilibrium with the Friesian allele, as described in Medugorac et al. [19]. Genotypes for candidate SNP g.1764239T>C and g.1855898G>A were inferred from genotypes at the other SNP. Of note, in both datasets, no recombination was observed within the Friesian haplotype between the Friesian candidate mutations or mutations showing strong linkage disequilibrium with them. In addition, neither the 80-kb duplication nor the SNP alleles associated with the *Polled* Friesian allele were found in p/p animals.

### Assessment of the Gene Content of the Polled Region

The gene content of the Celtic and Friesian *Polled* intervals was assessed based on the gene annotation available for the UMD3.1 genome assembly in the genome browser of the University of California, Santa Cruz (UCSC) (USA) (http://genome.ucsc.edu/).

### Analysis of Sequence Conservation around the Celtic Mutation using Multispecies Alignment

Region encompassing the Celtic mutation was PCR amplified from genomic DNA of American bison (*Bison bison*), water buffalo (*Bubalus Bubalis*), Nilgai (*Boselaphus tragocamelus*), blackbuck antelope (*Antilope cervicapra*), Siberian ibex (*Capra sibirica*), Wild goat (*Capra aegagrus*), Siberian bighorn sheep (*Ovis nivicola*), and fallow deer (*Dama dama*), and bidirectionally sequenced using the Sanger method, as previously described. These DNA samples are part of the INRA GABI unit DNA collection. The corresponding region of alpaca (*Vicugna pacos*) genome was obtained by BLATing to the bovine sequence the draft alpaca genome with the University of California, Santa Cruz (UCSC) genome browser (http://genome.ucsc.edu/). Multispecies alignment was generated with ClustalW software, version 2.0.1 [87].

### Histological Preparation

Tissues were fixed in paraformaldehyde (4%) at +4°C and subsequently dehydrated in a graded ethanol series, cleared with xylene and embedded in paraffin. Microtome sections (5 µm, Leica RM2245) were stained with haematoxylin, eosin and saffron (HES). Digital images were obtained with the NanoZoomer 2.0-HT slide scanner (Hamamatzu).

### Quantitative RT-PCR

RNA was extracted using the RNeasy Mini Kit (Qiagen). Super-Script II (Invitrogen) was used to synthesize cDNA from 2 µg of total RNA isolated from 90 dpc fetal tissues (horn bud and frontal bone, forehead skin and frontal bone). Bovine gene sequences were obtained from the UCSC genome browser, and PCR primers (Table S4) were designed using Primer Express Software for Real-Time PCR 3.0 (Applied Biosystems). Primer efficiency and specificity were evaluated on bovine genomic DNA. Quantitative PCR was performed in triplicate with 2 ng of cDNA (or 20 ng if the cycle threshold was too low with 2 ng) using the Absolute Blue SYBR Green ROX mix (Thermo Fisher Scientific) and the StepOnePlus Real-Time PCR System (Applied Biosystems). Results were analyzed with Qbase software using three appropriate normalizing genes (*RPLP0*, *HPRT1* and *GAPDH*).

## Supporting Information

Figure S1 **Details of the abnormal prepuce withdrawal phenotype displayed by some polled bulls.** Genital tracts of a horned Charolais bull (A) and of two P_C_/p Charolais bulls during penis withdrawal.
(TIF)

Table S1 **Candidate causative mutations for Cha-PH1 and Hol-PH1 based on whole genome sequencing data.** *The duplicated segment differs from the original by two mutations corresponding to g.1909354T>A and g.1909390_1909391del based on the reference sequence. The unique candidate causative mutation for the polled Celtic allele and the five candidate causative mutations for the polled Friesian allele according to Medugorac et al. (2012) are in italics and bold type, respectively.
(DOC)

Table S2 **Distribution of the Celtic and Friesian alleles in a large panel of polled and horned animals.** a: Central European horned breeds with polled strain traced back to the beginning of the 19^th^ century. b: Western European breed with polled strain traced back to the end of the 19^th^ century [88]. c: zebu and zebu X taurine African breeds.
(DOC)

Table S3 **Details of animals and designs used in the different experiments.** Breed abbreviations: AAN: Aberdeen-Angus, ABO: Abondance, AUB: Aubrac, BAQ: Blonde d’Aquitaine, BAZ: Bazadaise, BSW: Brown Swiss, CHA: Charolais, FJL: Fjäll, FSI: French Simmental, GAN: German Angus, GAS: Gasconne, GFV: German Fleckvieh, GLW: Galloway, HOL: Holstein, ICL: Icelandic Cattle, LIM: Limousine, MON: Montbéliarde, NOR: Normande, ORO: Östnorsk Rödkulla, PAR: Parthenaise, ROU: Rouge des prés (Maine-Anjou), RDA: Red Danish, SAL: Salers, SKB: Svensk kullig boskap, SRO: Svensk Rödkulla, TAR: Tarentaise, VOS: Vosgienne, VRO: Västnorsk Rödkulla, WAG: Wagyu. Species abbreviations: Bta: *Bos taurus* (cattle, wild-type allele), Bbi: *Bison bison* (American bison), Bbu: *Bubalus Bubalis* (water buffalo), Btr: *Boselaphus tragocamelus* (Nilgai), Ace: *Antilope cervicapra* (blackbuck antelope), Cae: *Capra aegagrus* (wild goat), Csi: *Capra sibirica* (Siberian ibex), Oni: *Ovis nivicola* (Siberian bighorn sheep), Dda: *Dama dama* (fallow deer), and Vpa: *Vicugna pacos* (alpaca). P^C^, P^F^ and p: Celtic polled, Friesian polled and horned alleles of the *Polled* locus.
(DOC)

Table S4 **Details on primers used in this study.**
(DOC)

Document S1 **Information on sequences replacing the UMD3.1 genome assembly gaps within the Friesian interval.**
(DOC)

Document S2 **Multispecies alignment of genomic sequences encompassing the Celtic mutation in** ***Bovidae*** **and non-** ***Bovidae*** **ruminant species.** Bta: *Bos taurus* (cattle, wild-type allele), Bbi: *Bison bison* (American bison), Bbu: *Bubalus Bubalis* (water buffalo), Btr: *Boselaphus tragocamelus* (Nilgai), Ace: *Antilope cervicapra* (blackbuck antelope), Cae: *Capra aegagrus* (wild goat), Csi: *Capra sibirica* (Siberian ibex), Oni: *Ovis nivicola* (Siberian bighorn sheep), Dda: *Dama dama* (fallow deer), and Vpa: *Vicugna pacos* (alpaca). The duplicated and deleted segments in the Celtic mutation are underlined and highlighted in yellow, respectively.
(DOC)
