# Supplementary figures and images for: Novel Insights into the Bovine Polled Phenotype and Horn Ontogenesis in Bovidae
Source: PLoS One. 2013 May 22;8(5):e63512. doi: 10.1371/journal.pone.0063512 (PMC3661542; doi:10.1371/journal.pone.0063512)

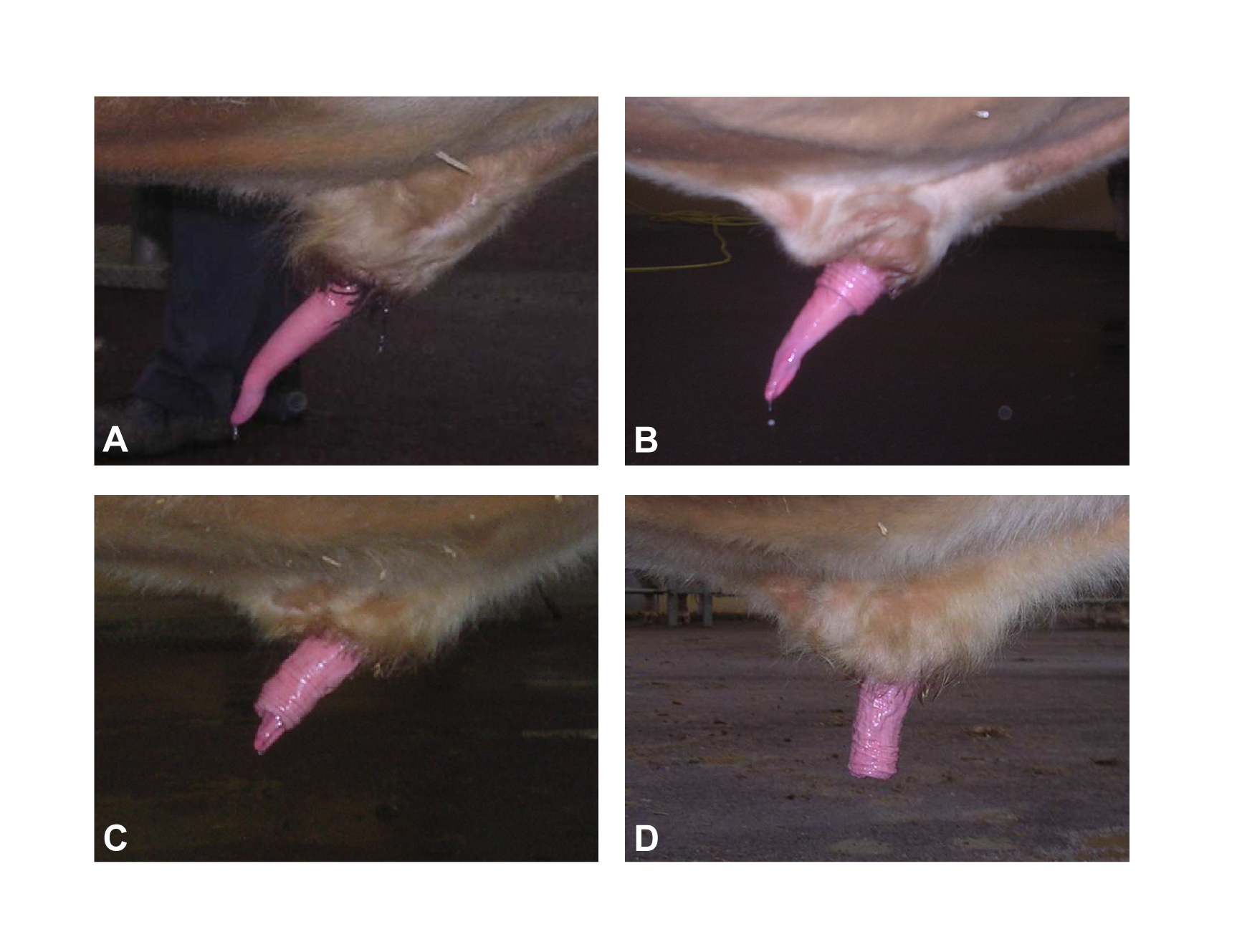

Supplement: Figure S1 — Details of the abnormal prepuce withdrawal phenotype displayed by some polled bulls. Genital tracts of a horned Charolais bull (A) and of two PC/p Charolais bulls during penis withdrawal. (TIF) [file pone.0063512.s001.tif]
