# Supplementary material for: Novel Insights into the Bovine Polled Phenotype and Horn Ontogenesis in Bovidae
Source: PLoS One. 2013 May 22;8(5):e63512. doi: 10.1371/journal.pone.0063512 (PMC3661542; doi:10.1371/journal.pone.0063512)
Supplement: Table S1 — Candidate causative mutations for Cha-PH1 and Hol-PH1 based on whole genome sequencing data. *The duplicated segment differs from the original by two mutations corresponding to g.1909354T>A and g.1909390_1909391del based on the reference sequence. The unique candidate causative mutation for the polled Celtic allele and the five candidate causative mutations for the polled Friesian allele according to Medugorac et al. (2012) are in italics and bold type, respectively. (DOC) [file pone.0063512.s002.doc]

| **Polymorphism** | **Associated polled haplotype** | **Polymorphism** | **Associated polled haplotype** |
| --- | --- | --- | --- |
| g.1389832_1389833 insT | Hol-PH1 | g.1635514G>C | Hol-PH1 |
| g.1389836_1389837insT | Hol-PH1 | g.1636329G>T | Hol-PH1 |
| g.1395536_1395537insA | Hol-PH1 | g.1639632A>G | Hol-PH1 |
| g.1400851_1400852del | Hol-PH1 | g.1640658G>T | Hol-PH1 |
| g.1402604G>A | Hol-PH1 | g.1640815T>C | Hol-PH1 |
| g.1407804C>A | Cha-PH1 | g.1642525T>A | Hol-PH1 |
| g.1521802T>A | Cha-PH1 | g.1645083G>T | Hol-PH1 |
| g.1552206G>A | Hol-PH1 | g.1645297del | Hol-PH1 |
| g.1556039C>T | Hol-PH1 | g.1645301T>A | Hol-PH1 |
| g.1583414A>G | Hol-PH1 | **g.1649163_1649169del-**  **insTTCTCAGAATAG** | Hol-PH1 |
| g.1587918TA>T | Hol-PH1 | g.1649330G>C | Cha-PH1 |
| g.1596777G>T | Hol-PH1 | g.1651491C>T | Hol-PH1 |
| g.1603747C>T | Hol-PH1 | **g.1654405G>A** | Hol-PH1 |
| g.1606620C>T | Hol-PH1 | **g.1655463C>T** | Hol-PH1 |
| g.1607675G>A | Hol-PH1 | g.1684055G>C | Hol-PH1 |
| g.1618261T>C | Hol-PH1 | *g.1706051_1706060del-*  *ins1705834_1706045dup* | Cha-PH1 |
| g.1623157G>A | Hol-PH1 | g.1764239T>C | Hol-PH1 |
| g.1624279C>T | Hol-PH1 | **g.1768587C>A** | Hol-PH1 |
| g.1624522T>G | Hol-PH1 | g.1833014A>C | Cha-PH1 |
| g.1624555T>C | Hol-PH1 | g.1855898G>A | Hol-PH1 |
| g.1626030T>C | Hol-PH1 | **g.1909352_1989480dup*** | Hol-PH1 |
| g.1626065G>C | Hol-PH1 | g.2028953C>G | Hol-PH1 |
| g.1626762T>C | Hol-PH1 | g.2089509C>T | Cha-PH1 |
| g.1628090A>G | Hol-PH1 | g.2180090C>T | Cha-PH1 |
| g.1631299A>G | Hol-PH1 | g.2381831T>C | Cha-PH1 |
| g.1631667C>T | Hol-PH1 | g.2383028G>A | Cha-PH1 |
| g.1631762G>A | Hol-PH1 | g.2387307G>A | Cha-PH1 |
| g.1631895T>C | Hol-PH1 | g.2388659A>G | Cha-PH1 |
| g.1633226T>A | Hol-PH1 | g.2393586_2393587insA | Cha-PH1 |
| g.1633690C>T | Hol-PH1 |  |  |
