# Supplementary material for: Novel Insights into the Bovine Polled Phenotype and Horn Ontogenesis in Bovidae
Source: PLoS One. 2013 May 22;8(5):e63512. doi: 10.1371/journal.pone.0063512 (PMC3661542; doi:10.1371/journal.pone.0063512)
Supplement: Table S2 — Distribution of the Celtic and Friesian alleles in a large panel of polled and horned animals. a: Central European horned breeds with polled strain traced back to the beginning of the 19th century. b: Western European breed with polled strain traced back to the end of the 19th century [88]. c: zebu and zebu X taurine African breeds. (DOC) [file pone.0063512.s003.doc]

| **Gr.** | **Breed** | **P_F_/P_F_** | **P_F_/P_C_** | **P_C_/P_C_** | **P_F_/p** | **P_C_/p** | **p/p** | **Total** |
| --- | --- | --- | --- | --- | --- | --- | --- | --- |
| Nordic breeds | Icelandic cattle | 0 | 0 | 9 | 0 | 3 | 0 | **12** |
|  | Fjäll | 0 | 0 | 12 | 0 | 0 | 0 | **12** |
|  | Norwegian Red | 0 | 0 | 1 | 0 | 6 | 13 | **20** |
|  | Östnorsk Rödkulla | 0 | 0 | 2 | 0 | 0 | 0 | **2** |
|  | Svensk Kullig Boskap | 0 | 0 | 6 | 0 | 0 | 0 | **6** |
|  | Svensk Rödkulla | 0 | 0 | 5 | 0 | 0 | 0 | **5** |
|  | Svensk x Östnorsk Rödkulla | 0 | 0 | 6 | 0 | 0 | 0 | **6** |
|  | Västnorsk Rödkulla | 0 | 0 | 1 | 0 | 0 | 0 | **1** |
|  |  |  |  |  |  |  |  |  |
| British breeds | Aberdeen Angus | 0 | 0 | 3 | 0 | 0 | 0 | **3** |
|  | British White | 0 | 0 | 1 | 0 | 0 | 0 | **1** |
|  | Irish Moiled | 0 | 0 | 1 | 0 | 0 | 0 | **1** |
|  | Galloway | 0 | 0 | 58 | 0 | 0 | 0 | **58** |
|  | Hereford | 0 | 0 | 29 | 0 | 10 | 3 | **42** |
|  | Highland cattle | 0 | 0 | 0 | 0 | 0 | 10 | **10** |
|  | White Galloway | 0 | 0 | 1 | 0 | 0 | 0 | **1** |
|  | Welsh Black | 0 | 0 | 0 | 0 | 1 | 1 | **2** |
|  |  |  |  |  |  |  |  |  |
| a | Beef Fleckvieh | 0 | 0 | 116 | 0 | 92 | 1 | **209** |
|  | Pinzgauer cattle | 0 | 0 | 3 | 0 | 11 | 42 | **56** |
|  |  |  |  |  |  |  |  |  |
| b | Holstein-Friesian | 33 | 4 | 0 | 179 | 41 | 251 | **508** |
|  |  |  |  |  |  |  |  |  |
| Horned breeds recently introgressed and synthetic breeds | Aubrac | 0 | 1 | 4 | 2 | 3 | 15 | **25** |
|  | Blanc Bleu Belge | 0 | 0 | 0 | 0 | 2 | 69 | **71** |
|  | Blonde d'Aquitaine | 0 | 0 | 2 | 0 | 3 | 23 | **28** |
|  | Bonsmara | 0 | 0 | 1 | 0 | 5 | 4 | **10** |
|  | Braunvieh | 0 | 0 | 1 | 0 | 7 | 79 | **87** |
|  | Charolais | 2 | 13 | 48 | 15 | 111 | 21 | **210** |
|  | Gelbvieh | 0 | 0 | 3 | 0 | 1 | 4 | **8** |
|  | German Angus | 0 | 0 | 94 | 0 | 3 | 0 | **97** |
|  | German Fleckvieh | 2 | 9 | 88 | 14 | 107 | 239 | **459** |
|  | Hinterwälder | 0 | 0 | 0 | 0 | 5 | 0 | **5** |
|  | Jersey | 1 | 1 | 1 | 4 | 4 | 41 | **52** |
|  | Limousin | 0 | 2 | 119 | 0 | 195 | 32 | **348** |
|  | Uckermärker | 0 | 0 | 13 | 0 | 10 | 2 | **25** |
|  | Vorderwälder | 0 | 0 | 0 | 0 | 4 | 16 | **20** |
|  | Witrug | 0 | 0 | 0 | 2 | 1 | 0 | **3** |
|  |  |  |  |  |  |  |  |  |

| **Gr.** | **Breed** | **P_F_/P_F_** | **P_F_/P_C_** | **P_C_/P_C_** | **P_F_/p** | **P_C_/p** | **p/p** | **Total** |
| --- | --- | --- | --- | --- | --- | --- | --- | --- |
| Traditionnally horned European, African and Asian taurine breeds | Abondance | 0 | 0 | 0 | 0 | 0 | 12 | **12** |
|  | Anatolian black | 0 | 0 | 0 | 0 | 0 | 36 | **36** |
|  | Barrosã | 0 | 0 | 0 | 0 | 0 | 14 | **14** |
|  | Baoulé | 0 | 0 | 0 | 0 | 0 | 7 | **7** |
|  | Bazadaise | 0 | 0 | 0 | 0 | 0 | 12 | **12** |
|  | Gasconne | 0 | 0 | 0 | 0 | 0 | 12 | **12** |
|  | Glenvieh | 0 | 0 | 0 | 0 | 0 | 1 | **1** |
|  | Istrian Cattle | 0 | 0 | 0 | 0 | 0 | 40 | **40** |
|  | Lekbibaj Cattle | 0 | 0 | 0 | 0 | 0 | 37 | **37** |
|  | Limpurger | 0 | 0 | 0 | 0 | 0 | 3 | **3** |
|  | Maronesa | 0 | 0 | 0 | 0 | 0 | 14 | **14** |
|  | Murnau-Werdenfelser | 0 | 0 | 0 | 0 | 0 | 3 | **3** |
|  | Podolian Cattle | 0 | 0 | 0 | 0 | 0 | 49 | **49** |
|  | Prespa Cattle | 0 | 0 | 0 | 0 | 0 | 19 | **19** |
|  | Salers | 0 | 0 | 0 | 0 | 0 | 12 | **12** |
|  | Sayaguesa Cattle | 0 | 0 | 0 | 0 | 0 | 2 | **2** |
|  | Tarentaise | 0 | 0 | 0 | 0 | 0 | 12 | **12** |
|  | Wagyu | 0 | 0 | 0 | 0 | 0 | 3 | **3** |
|  |  |  |  |  |  |  |  |  |
| c | Dwarf Zebu (Sri-Lanka) | 0 | 0 | 0 | 0 | 0 | 14 | **14** |
|  | Mbororo Zebu | 0 | 0 | 0 | 0 | 0 | 3 | **3** |
|  | Madagascar Zebu | 0 | 0 | 0 | 0 | 0 | 12 | **12** |
|  | Nguni | 0 | 0 | 0 | 0 | 0 | 4 | **4** |
|  | Kouri | 0 | 0 | 0 | 0 | 0 | 3 | **3** |
|  | Borgou | 0 | 0 | 0 | 0 | 0 | 5 | **5** |
|  |  |  |  |  |  |  |  |  |
|  | **Total** | **38** | **30** | **628** | **216** | **625** | **1195** | **2732** |
|  |  |  |  |  |  |  |  |  |
