# Supplementary material for: Novel Insights into the Bovine Polled Phenotype and Horn Ontogenesis in Bovidae
Source: PLoS One. 2013 May 22;8(5):e63512. doi: 10.1371/journal.pone.0063512 (PMC3661542; doi:10.1371/journal.pone.0063512)
Supplement: Table S3 — Details of animals and designs used in the different experiments. Breed abbreviations: AAN: Aberdeen-Angus, ABO: Abondance, AUB: Aubrac, BAQ: Blonde d’Aquitaine, BAZ: Bazadaise, BSW: Brown Swiss, CHA: Charolais, FJL: Fjäll, FSI: French Simmental, GAN: German Angus, GAS: Gasconne, GFV: German Fleckvieh, GLW: Galloway, HOL: Holstein, ICL: Icelandic Cattle, LIM: Limousine, MON: Montbéliarde, NOR: Normande, ORO: Östnorsk Rödkulla, PAR: Parthenaise, ROU: Rouge des prés (Maine-Anjou), RDA: Red Danish, SAL: Salers, SKB: Svensk kullig boskap, SRO: Svensk Rödkulla, TAR: Tarentaise, VOS: Vosgienne, VRO: Västnorsk Rödkulla, WAG: Wagyu. Species abbreviations: Bta: Bos taurus (cattle, wild-type allele), Bbi: Bison bison (American bison), Bbu: Bubalus Bubalis (water buffalo), Btr: Boselaphus tragocamelus (Nilgai), Ace: Antilope cervicapra (blackbuck antelope), Cae: Capra aegagrus (wild goat), Csi: Capra sibirica (Siberian ibex), Oni: Ovis nivicola (Siberian bighorn sheep), Dda: Dama dama (fallow deer), and Vpa: Vicugna pacos (alpaca). PC, PF and p: Celtic polled, Friesian polled and horned alleles of the Polled locus. (DOC) [file pone.0063512.s004.doc]

| **Experiment** | **Homozygous Polled** | **Heterozygous Polled** | **Homozygous**  **horned** | **A priori unknown phenotype** | **Structure** |
| --- | --- | --- | --- | --- | --- |
| IBD mapping attempt of the polled locus using Illumina bovine 777K SNP beadchip genotyping data | 2 AAN, 1 CHA, 2 FJL, 1 HOL, 2 ICL, 1 ORO, 2 SKB, 2SRO and 2 VRO | 4 CHA, 4 HOL and 12 LIM | 52 VOS, 87 BAZ, 100 BSW, 126 FSI, 150 ROU, 161 GAS, 182 TAR, 199 ABO, 298 PAR, 243 SAL, 252 AUB, 327 BAQ, 473 HOL, 475 LIM, 528 MON, 535 NOR and 671 CHA |  | Unrelated animals (homozygous polled) and small paternal half-sib design with 3 to 6 progeny (heterozygous polled and horned animals) |
| Mapping of polled locus using Illumina bovine 50K SNP beadchip genotyping data |  |  |  | 3349 CHA and 76851 HOL | Large paternal and small maternal half-sib design (French Genomic selection database) |
| Identification of candidate causative mutations for the polled locus using Illumina Hiseq whole genome sequencing data | 1 CHA and 1 HOL |  | 1 CHA, 8 MON, 9 NO and 37 HOL |  | Unrelated animals |
| Genotyping candidate mutations for the Celtic allele on homozygous animals of Nordic and British | 2 AAN, 8 FJL,  8 ICL, 2 ORO,  6 SKB, 5 SRO and 1 VRO |  |  |  | Unrelated animals |
| Genotyping of candidate mutations for the Celtic and Friesian alleles on a large panel of animals. See Table S2.  Among the animals studied, 968 have been genotyped in the framework of a previous study [19] | | | | | Unrelated animals for most of the breeds |
| Studying across-species conservation of the wild-type alleles of the Celtic mutation.  Bta (one horned HOL and one horned CHA), 2 Bbi, 4 Bbu, 2 Btr, 2 Ace, 2 Cae, 2 Csi, 2 Oni, 2 Dda and Vpa (draft genome assembly) | | | | | Unrelated animals |
| Phenotyping for eyelash phenotype | P_C_/P_C_: 3 CHA, 8 GLW, 1 GFV, 1 GAN; P_F_/P_F_: 3 HOL | P_C_/p: 31 CHA, 6 HOL, 4 LIM, 3 GFV; P_F_/p: 17 HOL | p/p: 54 CHA, 78 HOL, 22 LIM, 10 GFV |  | Unrelated animals |
| Phenotyping for preputial phenotype | P_C_/P_C_: 2 CHA, 1 GLW, 1 GFV, 1 GAN; P_F_/P_F_: 1 HOL | P_C_/p: 14 CHA, 1 HOL, 3 GFV; P_F_/p: 17 HOL | p/p: 21 CHA, 36 HOL, 10 GFV |  | Unrelated animals |
