# Supplementary material for: Novel Insights into the Bovine Polled Phenotype and Horn Ontogenesis in Bovidae
Source: PLoS One. 2013 May 22;8(5):e63512. doi: 10.1371/journal.pone.0063512 (PMC3661542; doi:10.1371/journal.pone.0063512)
Supplement: Table S4 — Details on primers used in this study. (DOC) [file pone.0063512.s005.doc]

| Gene/Mutation/Region | Forward | Reverse | Purpose |
| --- | --- | --- | --- |
| C1H21orf62 | CCTTCAGGTCAGCGTAGAGC | AGAACCCGTCTTGGTCACTG | RT-qPCR |
| E-Cadherin | CCTGAAGTGACTCGCAATGA | AAAGTTTCCAATTTCATCAGGA |
| N-Cadherin | GCAGCCTGATACGGTAGAGC | AGATCGAACCGGGTACTGG |
| FOXC2 | AACGAGTGCTTCGTCAAGGT | AGCTGCCGTTCTCGAACAT |
| FOXL2 | CCGGCATCTACCAGTACATTATAGC | GCACTCGTTGAGGCTGAGGT |
| GAPDH | CACTACCATGGAGAAGGCTGG | GTGGTTCACGCCCATCACA |
| GART | GCCCGAGTACTTGTCATTGG | CCTGGGGTAACCAACACTTG |
| HPRT1 | GAACGGCTGGCTCGA | TCCAACAGGTCGGCAAAGAA |
| IL10RB | TCTGTGATGACGCTCAGTTTG | TTTATGAGCCACCCTCATCAC |
| IFNAR1 | TCTTGACAGACTCACTGCTGCT | GAAGCAAATCTGAAGCCTGAA |
| IFNAR2 | CTCAAGGGCTCTCACACACA | ACTTCCGAGGAGGACAGTGA |
| IFNGR2 | CTTGGACCAGCACTTTGGTT | CTCGCCAAAGGATGACACTT |
| LincRNA#1 | ACCAGGAGGGGAGAAAGAAA | TTCGGGAGAGGAAGGAGGT |
| Occludin | CCGGAAGATGAAATTCTCCA | GCGATGCACATCACGATAAC |
| OLIG1 | CATCATCGCGACAAAACATC | AATTCCCAGGTCGATGAGTG |
| OLIG2 | TTCACAGCAACATCCTTTGAG | TACCCCCTTTTTCCGTCTCT |
| RPLP0 | TCTCCTTCGGGCTGGTCAT | AGGAAGCGGGAATGCAGAGT |
| RXFP2 | AGAACCCCACAATCCAGATG | TGACCCTGGAGAAGTTCCTG |
| TMEM50B | TGGCAGGCTTCCTAGATAATTT | CTGCAACCACAGAAGCCACT |
| TWIST1 | TGTGAGTCAGTTTGATCCCAAT | GGCATCATTATGGACTTTCTCC |
| TWIST2 | TTCAACTGGACCAAGGCTCT | CCAGGCTAACTTGAGACAGTCA |
| Vimentin | TGAAGGAAGAGATGGCTCGT | TCCAGCAGCTTCCTGTAGGT |
| ZEB2 | ACACGGGTTCTGAAACTGATGA | GGCACGCTAGCTGGACTTCT |
| g.1654405G>A | CGCTCCAAATTCATACGTCA | TGAACTGAACTTACAGGTGAAGGA | Genotyping of candidate polymorphisms for the Friesian allele |
| g.1655463C>T | TTCCTATTGTGCCAGGTGTG | ACGAGAAGGAGGGTGAGGTC |
| g.1684055G>C | ACACGACTGAGCGACAACTG | TTCCCAGGTCAGTGTGATCC |
| g.1764239T>C | ATCTCCCAGTGGTGAGTTGC | CCATGCGTGCATACATTTTC |
| g.1768587C>A | CCACGGATTACACAGTCGTG | CTACCTCCCTTGCCATTCAA |
| g.1855898G>A | TCCAGCAATGAGACAAAATG | TCCATCTAATCTCCCCCTGTT |
| 80-kb duplication | CCATCTTGGGTACAGCGTTT | GGCCCTGGAAGAAACCTACA |
| 80-kb control region | AGGGAAGCAGCTGAGATGAA | ACCAAGGTGCTCTGGTGTTT |
| g.2028953C>G | GCTCCCTGTGTTACAGAGCA | ATTTCCATGGCAACCAGAAG |
| g.1407804C>A | CGACCTTGGCTCAGAGGA | TCCAACGGATAAAAGCCAAC | Genotyping of candidate polymorphisms for the Celtic allele |
| g.1521802T>A | AAAAGAACTCCCCGAGTTGG | GCACACAGCACTTGCTCATT |
| g.1649330G>C | GCAGAAATGAAACATCTGTCCA | ATTCCCTCGGCTTTACGACT |
| g.1706051_1706060del-ins1705834_1706045dup | GAAGTGTGGCCGGTAGAAAA | ATCAAGGACACCTCCCACAC |
| g.1833014A>C | ATTTTCCCCCTTGTCAAACC | ATGCATTTGAGGGGACTGAG |
| g.2089509C>T | TGACACTGGGACAGAATCTCC | CTTTCCCTCCCCCAAAATTA |
| g.2180090C>T | TTGCGTTTTCTCAGGTGATG | ACGGGTTCTGACCATAATGC |
| g.2381831T>C | CACAGTCAATCCCACAGACG | AGGTCCTTGGTAAGGCAACC |
| g.2383028G>A | CTTCCGTCGACTTTTCTCCA | ACTCGCAGGGAAACTGTCAC |
| g.2387307G>A | GGTCCAGCTCCATGATGATT | CCTTCCTCACAGGCTCAAAG |
| g.2388659A>G | TTGGGTTCGAAAGGATCTTG | CTAGGAAGAGCCGGAGAAGG |
| g.2393586_2393587insA | CACCAGCCTTTCCATCATCT | CAAGCCACGGTAACCAAAGT |
| Region encompassing the wild-type Celtic allele | TCTGAGCTCACGCACAGC | GGCAGAGATGTTGGTCTTGG | Analysis of accross-species conservation |
| Gap located from position 1,704,428 bp to 1,704,522 bp | TTTGGATAGGGCTGAGCTGT | TCAGCGATGTATGAGGGAAA | Sequencing of gaps in the UMD3.1 assembly within the Friesian interval |
| Gap located from position 1,739,719 bp to 1,740,580 bp | TTCATTTGGGGAGAAACTGG | GGCCCATTTGACTCTGATGT |
| Gap located from position 1,780,403 bp to 1,780,502 bp | GTCCTGGGGGTGTCTGAGTA | TTACCTGCTTGAGGGGTCTTT |
| Gap located from position 1,824,554 bp to 1,824,653 bp | CCAGAGCAAATGCAACCTCT | AACTCGCTGGAGGAGATGAA |
| Gap located between position 1,867,486 bp and 1,867,585 bp due to a partial duplication and an error in genome assembly | CCAAGGCCAGTTGTTTCATT | GGTGGAAAAAGGCAGTGTGT |
