# Supplementary material for: Novel Insights into the Bovine Polled Phenotype and Horn Ontogenesis in Bovidae
Source: PLoS One. 2013 May 22;8(5):e63512. doi: 10.1371/journal.pone.0063512 (PMC3661542; doi:10.1371/journal.pone.0063512)
Supplement: Document S1 — Information on sequences replacing the UMD3.1 genome assembly gaps within the Friesian interval. (DOC) [file pone.0063512.s006.doc]

>Sequence replacing the polyN stretch located from position 1,704,428 bp to 1,704,522 bp TCCATGGTGTCACACAGAGTCAGACACAACTGAGTGACTGAGCACACACGGATGCAG

>Sequence replacing the polyN stretch located from position 1,739,719 bp to 1,740,580 bp

AGACGGTGGCATTTTTCTACGCAATTATTCATTCTTCCTTTCCCTCTCTTTCTGTGTATTTAGTAAGTACAGCATTGTATAGTATAGCATGATTCTAGGCAGAACACATCCCCTCATTAGAATTACATTATCTCACATTCTAGGAGGGGAATCAAGGCTCAGG

>Sequence replacing the polyN stretch located from position 1,780,403 bp to 1,780,502 bp

GAGAGGAAGAGGCCTCAGGAAGGATCTGAGTCCCCTGCAGAATTGTATGGACAAGAGGGAGAGAGATCAAGGAAATCACCTTAAAAGTAAACTCCGGG

> Sequence replacing the polyN stretch located from position 1,824,554 bp to 1,824,653 bp

GCGGGGGCACCTGGCACATGCTGCAGGGGCAGGGCATGCCGCCCCAGTGCTGGAAGCCGCCAGTGCCCCCGCCGCTGCCGCCCCCGCCCCCAAGCGGGGCCGCCGCCGCCGCCGCCGCCGGAGACTTGAGCAGGCCGTGAGGGGGCCGAATGGAGCCGACCGACGACAACCCGGAGCCGGGCAGGGAGGCGCTGGACACCGCGGCGGCCGCCGCGGCGGC

The polyN stretch located between position 1,867,486 bp and 1,867,585 bp is due to a partial duplication and an error in genome assembly. The whole region between positions 1866607 bp and 1868267 bp (excluded) must be excised and nucleotide 1866607 directly bound to nucleotide 1868267.
