# Supplementary material for: Novel Insights into the Bovine Polled Phenotype and Horn Ontogenesis in Bovidae
Source: PLoS One. 2013 May 22;8(5):e63512. doi: 10.1371/journal.pone.0063512 (PMC3661542; doi:10.1371/journal.pone.0063512)
Supplement: Document S2 — Multispecies alignment of genomic sequences encompassing the Celtic mutation in Bovidae and non- Bovidae ruminant species. Bta: Bos taurus (cattle, wild-type allele), Bbi: Bison bison (American bison), Bbu: Bubalus Bubalis (water buffalo), Btr: Boselaphus tragocamelus (Nilgai), Ace: Antilope cervicapra (blackbuck antelope), Cae: Capra aegagrus (wild goat), Csi: Capra sibirica (Siberian ibex), Oni: Ovis nivicola (Siberian bighorn sheep), Dda: Dama dama (fallow deer), and Vpa: Vicugna pacos (alpaca). The duplicated and deleted segments in the Celtic mutation are underlined and highlighted in yellow, respectively. (DOC) [file pone.0063512.s007.doc]

Bta -----AGAAGGCGGCACTATCTTGATGGAACTCAGTCTCATCACCTGTGAAATGAAGAGT

Bbi ----CAGAAGGCGGCACTATCTTGATGGAACTCAGTCTCATCACCTGTGAAATGAAGCGT

Bbu -CTCATGAAGGCGGCACTATCTTGATGGAACTCAGTCTCATCACCTGTGAAATGCAGAGT

Btr -CCTCAGAAGGCGGCACTATCTTGATGTTACTCAGTCTCATCACCTGTGAAATGAAGAAT

Cae -TTTCTGAAGGCGGCACTATCTTGATGGAGCTCATTCTCATCACCTGTGAA-TGAAGAGT

Csi ATATCAGAAGGCGGCACTATCTTGATGGAGCTCATTCTCATCACCTGTGAA-TGAAGAGT

Oni TCTTCAGAAGGCGGCACTATCTTGATGGAGCTCATTCTCATCATCTGTGAA-TGAAGAGT

Ace ---TCAGAAGGCGGCACTATCTTGATGGAGTTCATTCTCATCACCTGTGAA-TGAAGAGT

Dda ---TCAGAAGGCGGCACTATCTTGATGCAATTCAGTCTCATCACCTGTGAAATGAAGAGT

Vpa --TCAGGCAGGTGGCACTGTCTTGATGGAACTCAGTCTCATCACCTGTGAAATGAAGAGT

* *** ****** ******** *** ******** ******* ** ** *

Bta ACGTGGTACCAACTACTTTCTGAGCTCACGCACAGCTGGACGTCTGCGCCTTTCTTGTTA

Bbi ATGTGGTATCAACTACTTTCTGAGCTCACGCACAGCTGGACGTCTGCGCCTTTCTTGTTA

Bbu ATGTGGTACCAACTACTTTCTGAGCTCACGCACAGCTGGATGTCTGCGCCTTTTTTGTTA

Btr ATGTGGTACCAACTACTTTCTGAGCTCACGCACAGCTGGACGTCTGCGCCTTTTTTGTTA

Cae ATGCGGTACCAACCACTTTCTGA-CTCATGCACAGCTGCACGTCTGTGCCTTTTATGTTA

Csi ATGCGGTACCAACCACTTTCTGA-CTCATGCACAGCTGCACGTCTGTGCCTTTTATGTTA

Oni ATGCGGTACCAACCACGTTCTGA-CTCACGCACAGCTGCACGTCTGTGCCTTTTATGTTA

Ace GTGTGGTACCAGCCACTTTCTGAGCTCACGCACAGCTGCACGTCTGTGCCTTTTATGTTA

Dda ATGTGGTACCAACTACTTCCTGACCTCACACACAGCTGGA-GTCTGTGTCTTT--TGTTA

Vpa ATGTGGTCCCAACCACTTCCTAAGGTCACATACACTTGGACATCTATGTCTTTTATGTTA

* *** ** * ** * ** * *** *** ** * *** * **** *****

Bta TACTGCAGATGAAAACATTTTATCAGATGTTTGCCTAAGTATGGATTACATTTAAGATAC

Bbi TACTGCAAATGAAAACATTTTATCAGATGTTTGTCTAAGTATGGATTACATTTAAGATAC

Bbu TACCGCAAATGAAAACATTTTATCAGATGTTTGTCTAAGTATGGATTACATTTAAGATAC

Btr TACCGCAAATGAAAACATTTTATCAGATGTTTGCCTAAGTATGGATTACATTTAAGATAC

Cae TACCGAAAATGAAAACATTTTATCAGATGTTTGCCTAAGTATGGATTACTTTTAAGATAC

Csi TACCGAAAATGAAAACATTTTATCAGATGTTTGCCTAAGTATGGATTACTTCTAAGATAC

Oni TACCGCAAATGAAAACATTTTATCAGATGTTTGCCTAAGTATGGATTACATTTAAGATAC

Ace TACCGCAAATGAAAACATTTTATCAGATGTTTGCCTAAGTATGGATTAAATTTAAGATAC

Dda TACCGCAAATGAAAACATTTTATCAGATGTTTGCCTAAGTATGGATTACAGTTAAGATAC

Vpa GATTACTAATGAAAATCTTTTATCAGATGTTTGGTTAAGTATGGATTATATTTAAGATAC

* ******* **************** ************* ********

Bta ATATTT---TTCTTTCTTGTCTGAAAGTCTTTGTAGTGAGAGCAGGCTGGAATTATGTCT

Bbi ATATTT---TTCTTTCTTGTCTGAAAGTCTTTGTAGTGAGAGCAGGCTGGAATTATGTCT

Bbu ATATTT---TTCTTTCTTGTCTGAAAGTCTTTGTAGTGAGAGCAGGCTGGAATTATGTCT

Btr ATATTT---TTCTTTCTTCTCTGAAAGTCTTTGTAGTGAGAGCAGGCTGGAATTATGTCT

Cae ACATTT---TTCTTTCTTGTCTGAAAGTCTTTGTAGTGAGAGCAGGCTGGAATTATGTCT

Csi ACATTT---TTCTTTCTTGTCTGAAAGTCTTTGTAGTGAGAGCAGGCTGGAATTATGTCT

Oni ACATTT---TTCTTTCTTGTCTGAAAGTCTTTGTAGTGAGAGCAGGCTGGAATTATGTCT

Ace ACATTT---TTCTTTCTTGTCTGAAAGTCTTTGTAGTGAGAGCAGGCTGGAATTATGTCT

Dda ATATCT---TTCTTTCTTGTCTGAAAGTCTTTGCAGTGAGAGCAGGCTGGAATTATGTCT

Vpa CTATATATATTTTTTCTTGCCTGAAAATCGCTGCAGTGAGAGCAGGCTGGAATTATGTCT

** * ** ****** ****** ** ** **************************

Bta GGGGTGAGATAGTTTTCTTGGTAGGCTGGTATTCTTGCTCTTTAGATCAAAACTCTCTTT

Bbi GGGGTGAGATAGTTTTCTTGGTAGGCTGGTATTCTTGCTCTTTAGATCAAAACTCTCTTT

Bbu GGGGTGAGATAGTTTTCTTGGTAGGCTGGTTTTCTTGCTCTTTAGATCAAAACTCTCTTT

Btr AGGGTGAGATAGTTTTCTTGGTAGGCTGGTTTTCTTGCTCTTTAGATCAAAACTCTCTTT

Cae AGGGTAAGATAGTTTTCTTGGTAGGCTGGTTTCCTTGCTCTTTAGATCAAAACTCTCTT-

Csi AGAGTAAGATAGTTTTCTTGGTAGGCTGGTTTCCTTGCTCTTTAGATCAAAACTCTCTT-

Oni AGGGTAAGATAGTTTTCTTGGTAGGCTGGCTTTCTTGCTCTTTAGATCAAAACTCTCTT-

Ace AGGGTAAGATAGTTTTCTTGGTAGGCTGGTTTTCTTGCTCTTTAGATCAAAACTCTCTT-

Dda AGGGTGAGATAGTTTTCTTGGTAGGCTGGTTTTCTTGATCTTTAGATCAGAACTCTCTTT

Vpa GGGGTGAGATAATTCTCTTAGTGGGCTGTTTTTCTTGATCTT-AGACCAAAACTCTCATT

* ** ***** ** **** ** ***** * **** **** *** ** ******* *

Bta TCATTTTTAAGTCTATCCCAAAAGTGTGGGAGGTGTCCTTGA------

Bbi TCATTTTTAAGTCTATCCCAAAAGTGTGGGAGGTGTCCTTAA------

Bbu TCATTTTTAAGTCTATCCCAAAAGTGTGGGAGGTGTCCTTAAACAC--

Btr TCATATTTAAGTCTATCCCAAAAGTGTGGGAGGTGTCCTTAAAGAGAC

Cae -CATATTTCAGTCTATCCCCAAAGTGTGGGAGGTGTCCTTAA------

Csi -CATATTTCAGTCTATCCCCAAAGTGTGGGAGGTGTCCTTAA------

Oni -CATATTTCAGTCTATCCCAAAAGTGTGGGAGGTGTCCTTAA------

Ace -CATATTTCAGTCTATCCCAAAAGTGTGGGAGGTGTCCTTAA------

Dda TCATATTTCAGTCTATCCCAAAAGTGTGGGAGGTGTCCTTAA------

Vpa -TATAATA----------------------------------------

** *
